# An internet-based bioinformatics toolkit for plant biosecurity diagnosis and surveillance of viruses and viroids

**DOI:** 10.1186/s12859-016-1428-4

**Published:** 2017-01-11

**Authors:** Roberto A. Barrero, Kathryn R. Napier, James Cunnington, Lia Liefting, Sandi Keenan, Rebekah A. Frampton, Tamas Szabo, Simon Bulman, Adam Hunter, Lisa Ward, Mark Whattam, Matthew I. Bellgard

**Affiliations:** 1 Centre for Comparative Genomics, Murdoch University, Murdoch, WA 6150 Australia; 2 Plant Biosecurity Cooperative Research Centre, Canberra, ACT 2617 Australia; 3 Department of Agriculture and Water Resources, Mickleham, VIC 3064 Australia; 4 Ministry for Primary Industries, Wellington, New Zealand; 5 The New Zealand Institute for Plant Food and Research Limited, Better Border Biosecurity, Lincoln, 7608 New Zealand

**Keywords:** Bioinformatics, Plant biosecurity, Next generation sequencing, Plant viruses and viroids, Quarantine, viRNAs, Virus diagnosis, Yabi, Small RNA-Seq, Workflows

## Abstract

**Background:**

Detection and preventing entry of exotic viruses and viroids at the border is critical for protecting plant industries trade worldwide. Existing post entry quarantine screening protocols rely on time-consuming biological indicators and/or molecular assays that require knowledge of infecting viral pathogens. Plants have developed the ability to recognise and respond to viral infections through Dicer-like enzymes that cleave viral sequences into specific small RNA products. Many studies reported the use of a broad range of small RNAs encompassing the product sizes of several Dicer enzymes involved in distinct biological pathways. Here we optimise the assembly of viral sequences by using specific small RNA subsets.

**Results:**

We sequenced the small RNA fractions of 21 plants held at quarantine glasshouse facilities in Australia and New Zealand. Benchmarking of several de novo assembler tools yielded SPAdes using a kmer of 19 to produce the best assembly outcomes. We also found that de novo assembly using 21–25 nt small RNAs can result in chimeric assemblies of viral sequences and plant host sequences. Such non-specific assemblies can be resolved by using 21–22 nt or 24 nt small RNAs subsets. Among the 21 selected samples, we identified contigs with sequence similarity to 18 viruses and 3 viroids in 13 samples. Most of the viruses were assembled using only 21–22 nt long virus-derived siRNAs (viRNAs), except for one Citrus endogenous pararetrovirus that was more efficiently assembled using 24 nt long viRNAs. All three viroids found in this study were fully assembled using either 21–22 nt or 24 nt viRNAs. Optimised analysis workflows were customised within the Yabi web-based analytical environment. We present a fully automated viral surveillance and diagnosis web-based bioinformatics toolkit that provides a flexible, user-friendly, robust and scalable interface for the discovery and diagnosis of viral pathogens.

**Conclusions:**

We have implemented an automated viral surveillance and diagnosis (VSD) bioinformatics toolkit that produces improved viruses and viroid sequence assemblies. The VSD toolkit provides several optimised and reusable workflows applicable to distinct viral pathogens. We envisage that this resource will facilitate the surveillance and diagnosis viral pathogens in plants, insects and invertebrates.

**Electronic supplementary material:**

The online version of this article (doi:10.1186/s12859-016-1428-4) contains supplementary material, which is available to authorized users.

## Background

Increases in global trade and movement are placing significant pressure on post entry quarantine systems, with an increase in the frequency of incursions of pathogens causing the emergence of diseases and pests that are both difficult and costly to eradicate and control [1]. The challenge of maximising the benefits of global trade whilst minimising the negative impacts of biosecurity threats is one faced by most nations [2]. Historically, the geographical isolation of Australia and New Zealand, coupled with stringent quarantine screening measures, has provided protection from the introduction of exotic pests and pathogens that have the potential to harm human health, agriculture, the environment and the economy.

Plant biosecurity is defined as “a set of measures designed to protect crops from emergency plant pests at national, regional and individual farm level” [1, 3]. The diagnosis of viral pathogens is a crucial component of plant biosecurity surveillance, required to prevent the potential introduction of exotic plant viruses and viroids. Existing ‘specific’ serological and molecular detection methods such as enzyme-linked immunosorbent assay (ELISA), polymerase chain reaction (PCR), or nucleic acid spot hybridization, while highly sensitive, are limited by their ability to detect only known plant viruses/viroids. These methods lack the capacity to detect unknown, poorly characterised or highly variable viral pathogens [4, 5]. Furthermore the host range of many viral pathogens is not defined and known exotic viruses/viroids could be missed if these infect new plant species for which standard screening assays are not applied. If pathogens are not initially detected via these methods, more ‘investigational’ techniques may be applied, such as electron microscopy, host plant inoculation, or PCR using degenerate primers [5]. The time and effort taken to screen imported plants using these existing methods has a direct economic impact, with plants that are currently imported into Australia and New Zealand spending up to two years in quarantine (https://bicon.agriculture.gov.au/BiconWeb4.0).

Recent studies have demonstrated both the detection of viral pathogens and the identification of novel viruses by the deep sequencing of small RNAs (small RNA-Seq) of plant species [4–7]. RNA silencing is a natural anti-viral defence system present in plants, insects and invertebrates that recognise dsRNA viral genomes and/or viral intermediate sequences, and cleave them into small interfering RNAs (siRNA) of 21-24 nt in length [8]. These virus-derived siRNAs (viRNAs) accumulate in the small RNA fraction of host plants making it amenable to identify viruses through a next generation sequencing (NGS) approach, even at extremely low viral titres and in symptomless infections [9, 10]. Small RNA NGS screening of viral pathogens is more cost- and time-effective compared with current detection methods. The bottleneck for the uptake of NGS technology for routine surveillance and diagnosis of viral sequences is the lack of an automated bioinformatics pipeline that enables users to evaluate, scrutinize and modify all key steps of the analysis workflow including de novo assembly parameters with access to intermediate outputs [10, 11]. This is key to optimise and increase the confidence in the de novo assembled sequences with similarity to viral genomes.

Two such automated pipelines have recently been made available, one designed to detect known and novel viruses through de novo assembly [VirFind, 11], and the other, limited to extract viRNAs through mapping onto a priori known viral reference genomes [12]. VirFind is available as a web-based graphical front-end interface, with users completing a sequence submission form and uploading sequence files via the VirFind ftp server. Users are able to set their own parameters at several selected stages of the pipeline, such as choosing to map to a host reference genome, the expected value for BLASTN and BLASTX, or choosing to search conserved domains.

L Miozzi and V Pantaleo [12] developed a pipeline to extract viRNAs through the open source Galaxy web-based platform [13]. This pipeline uses a reference guided approach to map reads to reference viral or viroid genomes, therefore viRNAs that correspond to viruses or viroids not present in public databases will be missed. Users upload a fastq file, and filter the results of the mapping to specific viruses or viroids of interest. The resulting SAM file can then be downloaded, and visualised using the java standalone tool MISIS [14].

An automated, yet customisable bioinformatics pipeline for the detection and screening of viruses and viroids is required for the adoption of NGS technology by agencies without established bioinformatics expertise. Previously, we presented Yabi, an analysis workflow environment that is able to create and reuse workflows, as well as manage large amounts of raw and processed data in a secure and flexible environment [15]. Yabi is accessed via a simple ‘drag and drop’ web-based environment by researchers without bioinformatics expertise or through the Yabi command line for advanced users. Individual tools can be configured and easily incorporated into sophisticated workflows in real time. Importantly, comprehensive provenance for each workflow is kept, including input files and the parameters used for each tool, enabling researchers to track previous analyses performed and share optimised workflows with others. Files can be managed across different high performance computing storage infrastructures.

This study presents a novel automated internet-based bioinformatics toolkit for the detection of viruses and viroids utilising the online research environment Yabi. This toolkit offers users the flexibility to process small RNA-Seq samples using existing optimised workflows and/or to further customise available tools or add new tools into the web-based analytical environment. It is envisaged that this resource will significantly reduce post entry quarantine ongoing costs and quarantine lead times. Furthermore, the web-based bioinformatics toolkit is customized to meet quarantine expectations, facilitate collaborations and inform policy makers.

## Methods

### Sample collection, RNA extraction and NGS sequencing

Imported plants and positive control samples were grown in quarantine glasshouse facilities until sample collection. Plants were grown under natural lighting with a daytime temperature of approximately 22 °C. For each plant sample one or more leaves were collected prior to RNA extraction. Total RNA and/or small RNA enriched fraction (<200 bp) were extracted from approximately 10 mg of tissue using the mirVana microRNA isolation kit (Ambion, LifeTechnologies) following manufactures instructions. Collected samples were stored at -80 °C within quarantine facilities until shipped to the Beijing Genomics Institute (BGI, Hong Kong). Libraries were prepared using the TruSeq Small RNA Sample Prep Kit (Illumina) and sequenced with 50 bp single-end (SE) reads deep sequencing of collected small RNA samples (small RNA-Seq) on an Illumina HiSeq2000. We sequenced 21 quarantined plant samples (Additional file 1). Small RNA-Seq datasets has been submitted to the Short Read Archive (SRA) under the BioProject PRJNA325594.

### Selection of small RNA assembler and scaffolding tools

We compared Velvet [16], SPAdes [17], ABySS [18] and SOAPdenovo [19] assemblers using twelve selected small RNA-Seq samples collected from distinct plant species generated in this study (Additional file 1). We tested de novo assembly using individual kme*r* lengths of 15 (K15), 17 (K17), 19 (K19) and 21 (K21) as well as combined kmer sets of 15,17,19 (K15-17-19) and 15,17,19,21 (K15-17-19-21). Assembled contigs were further scaffolded using CAP3 using optimised parameters for short overlaps (-o 16, -p 90, -i 30, -j 31, -s 300) [20]. Additionally, merging and scaffolding of contigs produced by two or all three assemblers were also evaluated. Assembly statistics were calculated using the Quality Assessment Tool for Genome Assemblies (QUAST) [21].

### Overview of the automated viral diagnosis and surveillance toolkit

The viruses and viroids surveillance and diagnosis (VSD) bioinformatics toolkit was developed utilising Yabi [15], an open source internet-based analytical environment that allows for the customisation of tools and scripts into analysis workflows [22]. Yabi has five tabs, namely, ‘Jobs’, ‘Design’, ‘Files’, ‘Account’ and ‘Admin’ tabs, where the later is only visible to a person or group responsible for the maintenance and further customisation of the Yabi platform [15]. The ‘Jobs’ tab allows visualising and downloading results from prior jobs. The ‘Design’ tab enables re-use of existing optimised workflows, design of modified versions of existing workflows, and the construction of new analysis workflows. The ‘Files’ tab present files and directories of all available backend resources (i.e. HPC and/or cloud instances) to the user [15]. The ‘Account’ tab enables a user to easily modify their password information to their Yabi account. The ‘Admin’ tab facilitates the management and addition of new computational tools into the Yabi environment. New features of the Yabi platform include: i) save and share workflows; ii) fetch data from public repositories; iii) submission of processed data to specialised databases such as National or International Patient Registries; and iv) enables ‘bioinformatics on demand’ analyses through the deployment of cloud instance at the beginning of a computational workflow and its obliteration at the final step of the data processing and analysis workflow.

**Fig. 1:**
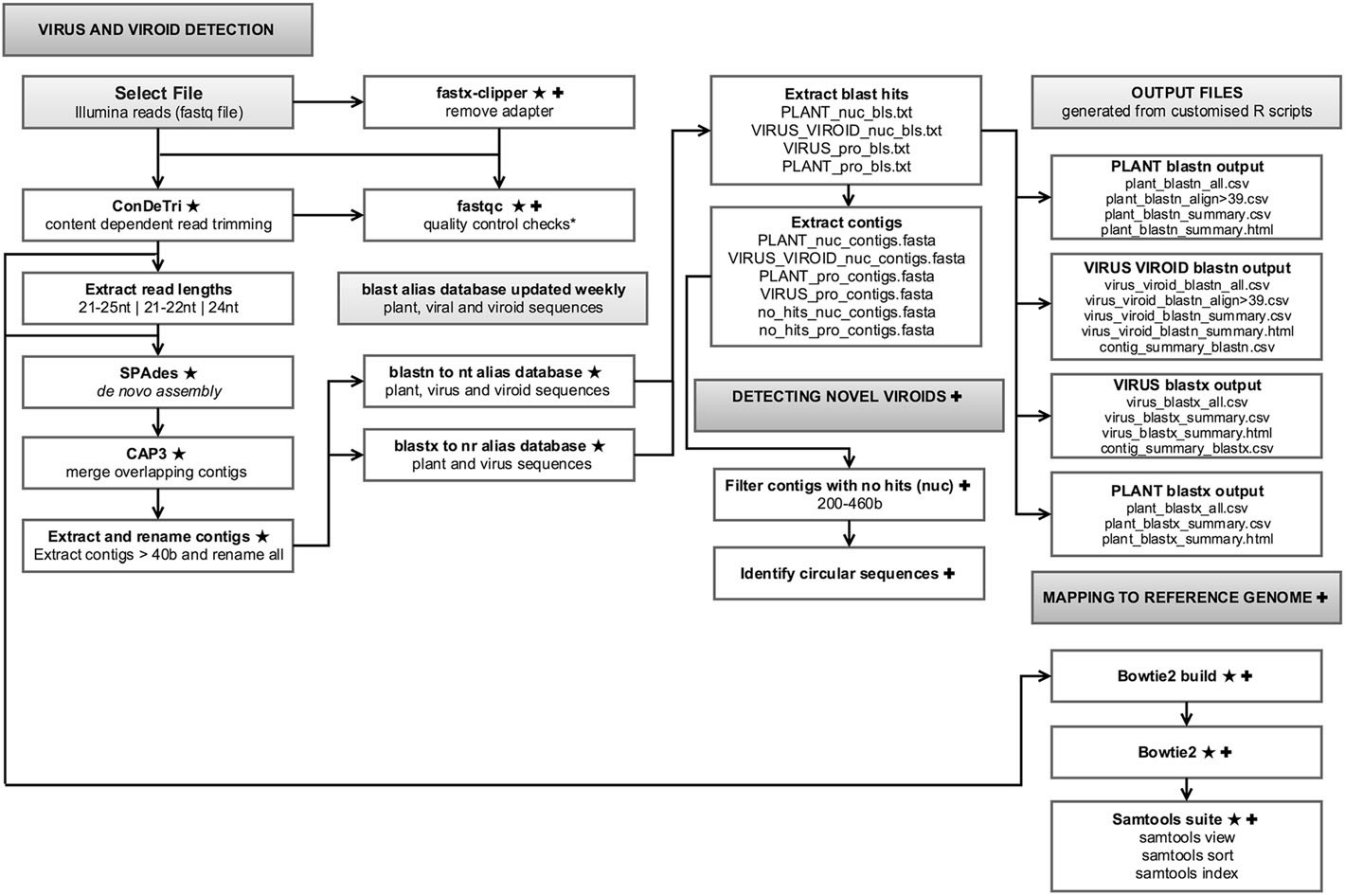
Workflow for the bioinformatics VSD toolkit for the discovery of viruses and viroids. Three versions of the workflow are available, with users able to choose from three options of read lengths (21–25 nt, 21–22 nt, or 24 nt length reads, ‘Extract read lengths’ tool). ★ indicates tools where users may change options or parameters as desired. ✚ indicates tools and workflows users can add or remove to the pipeline as by default these are not part of the automated VSD pipeline. ★ ✚ both ★ and ✚ options are available to users. *Results from this optional step are not used in downstream analyses

The VSD toolkit has three versions of the ‘virus and viroid detection’ workflows (Fig. 1), with users able to choose from three subsets of small RNA read lengths (21–25 nt, 21–22 nt, or 24 nt length reads). Existing automated workflows can be reused or modified and saved (Additional file 2). Additional workflows such as the ‘detecting novel viroids’ and ‘mapping reads onto a reference genome’ are also available, and can be run as a separate job, or added to the ‘virus and viroid detection’ workflows (Additional file 2).

#### Virus and viroid detection workflow

Files of small RNA reads in fastq format (gzipped files are accepted) are first uploaded through the ‘Files’ tab in Yabi. Files may be uploaded directly from a personal computer or transferred to a Yabi directory from another high performance computing storage location.

Once the file is uploaded, users then navigate to the ‘Design’ tab, where they choose the saved workflow of interest (21–25 nt, 21–22 nt or 24 nt length reads). Users are also able to build their own workflows, by simply dragging and dropping tools into the workflow area. The first stage of the workflow is the ‘Select file’ tool. The fastq file of interest is then selected. If an adapter trimming step needs to be performed, users can add in the ‘fastx_clipper’ tool (http://hannonlab.cshl.edu/fastx_toolkit/) to the workflow, and perform quality control checks using the ‘fastQC’ tool [23]. Reads then undergo quality trimming through the content dependent read trimming tool ConDeTri (version 2.2), which trims and removes reads with low quality scores [24]. Minimum read length is set to 18 nt. Reads of the desired read length are then extracted through the ‘Extract_reads_21–25 nt’ , ‘Extract_reads_21–22 nt’ , or ‘Extract_reads_24 nt’ tools with de novo assemblies of contigs performed with SPAdes (version 3.5.0) with kmer sizes set to 15,17 and 19 [17]. Overlapping SPAdes contigs are then merged with CAP3 (version date 08/06/13) [20].

Contigs greater than or equal to 40 nt are then extracted using an in-house python script (‘Extract and rename contigs’ tool), and BLAST searched against databases generated from all plant, virus and viroid sequences populated by the entrez search query’s for viruses “txid10239 [orgn]”, not cellular organisms “txid131567 [orgn]”, viroids “txid12884 [orgn]” and plants “txid3193 [orgn]”. For BLASTN, the task is set to BLASTN short, and for both BLASTN and BLASTX, the maximum number of aligned sequences (-max_target_seqs) is set to 5 and the expected value (-evalue) set to 1e^−10^. BLASTN and BLASTX results are written out in a customised tabular format and extracted through an in-house script (‘Extract BLAST hits’ tool) into ‘plant’ or ‘virus and viroid’ BLAST output files. Contigs are also extracted into fasta output files through an in-house script (‘Extract contigs’) into subsets with a BLAST hit to plant or virus and viroid sequences, and contigs with no BLAST hits. The BLAST output files are then parsed through an in-house script, which produces several output files in csv format including all blast results in tabular format (header added), blast results with alignment lengths >39 nt, and a summary file which reports the Genbank ID of the virus or plant hit, the name of the plant or virus, the number of contig hits, the average percent sequence identity of the hit to the virus, the alignment length, the length of the virus or plant sequence, and the percentage coverage by contigs of a virus or plant sequence. These statistics are calculated using the Bioconductor’s GenomicRanges package (version 1.18.1).

#### Detecting novel viroids workflow

The output file ‘no_hits_contigs.fasta’ from the ‘Extract contigs’ tool represent sequences with no BLASTN and BLASTX sequence similarity to viral and plant sequences. These sequences are further filtered to extract contigs with lengths between 200–460 b that are typical for viroids. An in-house script is then utilised to evaluate the sequence similarity and overlap of both 5′-end and 3′-end of selected sequences. Sequences with overlapping ends with 100% sequence similarity are reported as putative circular viroid candidates.

Users are recommended to further inspect identified candidate circular sequences. For example evaluate sequence similarity to non-coding RNA databases such as Rfam [25] and miRBase [26], which are not part of the VSD toolkit. Viroids from the Pospiviroidae (e.g. *Grapevine yellow speckle viroid 1*) and some from the Avsunviroidae (e.g. *Avocado sunblotch viroid*) form hairpin-like RNA secondary structures [27]. Thus, such confirmation can be evaluated in filtered candidate novel viroid circular sequences using RNAfold [28]. Furthermore, the expression of candidate sequences passing all filtering steps can be evaluated in multiple tissues of the infected plant and/or its progeny to validate i) the de novo assembled circular sequence, and ii) provide independent evidence of its expression in multiple tissues and/or individuals.

#### Mapping small RNAs onto reference genome workflow

Mapping of viRNAs onto identified viral genomes from similarity searches typically provide a broader coverage of the viral pathogen sequence as compared to de novo assembled contigs. Quality trimmed reads or the subset of reads (21–25 nt, 21–22 nt, or 24 nt length reads) can be mapped against a reference genome of choice (fasta file must also be uploaded by users) through bowtie2 [29]. Optionally, the SAMtools suite (Fig. 1) [30] can be used to sort and index aligned reads. The resulting alignment file (in sam or bam format) can then be downloaded and viewed using the java standalone tool MISIS [14].

#### Unique features of the VSD toolkit

The major unique feature of our VSD toolkit as compared to VirFind [11], is the ability to exclusively use 21–22 nt small RNA reads for the de novo assembly of viral sequences. Assembly of viral sequences with this set of reads directly reflect the plant endogenous antiviral response mediated by Dicer4 and Dicer2 [31]. Additionally, we provide an assembly pipeline that uses 24 nt small RNAs overlapping the expected size for endogenous heterochromatin and transposon derived siRNAs [32]. This 24 nt pipeline identifies viral sequences potentially integrated in the host genome, particularly if they are not detected using 21–22 nt pipeline. Finally, we provide a 21–25 nt pipeline for users to compare their outputs against other published work that typically use a broad range of small RNAs and/or compare with the results from the targeted 21–22 nt and 24 nt pipelines.

Another unique feature of the VSD toolkit is the ability to modify the parameter options for most of the individual steps in the workflow (Fig. 1). Additionally, the VSD toolkit uses optimised SPAdes de novo assembly settings that yield improved results as compared to other tested assemblers (See below).

The similarity screening of viruses in VSD toolkit is run in parallel using both BLASTN and BLASTX [33] for all de novo assembled contigs against viruses and viroid sequences in the NT and NR databases (ftp://ftp.ncbi.nlm.nih.gov/blast/db/), respectively. The top five database hits for each de novo assembled contig are reported improving coverage of specific isolate/strain viral sequences and/or preventing false negative results when a top viral hit is annotated as “synthetic sequence”. VirFind runs BLAST screening in a staggered manner, reporting first nucleotide top hits against viral sequences, and then for contigs with negative BLASTN results, a BLASTX screening is conducted reporting the best hit [11].

Finally, the VSD toolkit also provides a list of potential viroid-like circular sequences with no sequence similarity to any nucleotide sequence in public databases.

#### Deployment of the toolkit

During optimisation and testing of the bioinformatics toolkit, the workflows were run on a dynamic SGE cluster located on Amazon Web Services (AWS) Elastic Compute Cloud (EC2), which allows compute nodes (29.4 GB RAM) to be easily added or removed as required. Yabi and the bioinformatics toolkit may be deployed on a variety of high performance computing resources. The source code is available from https://github.com/muccg/yabi.

## Results and discussion

### Selection of de novo assembler for viral sequences

**Fig. 2:**
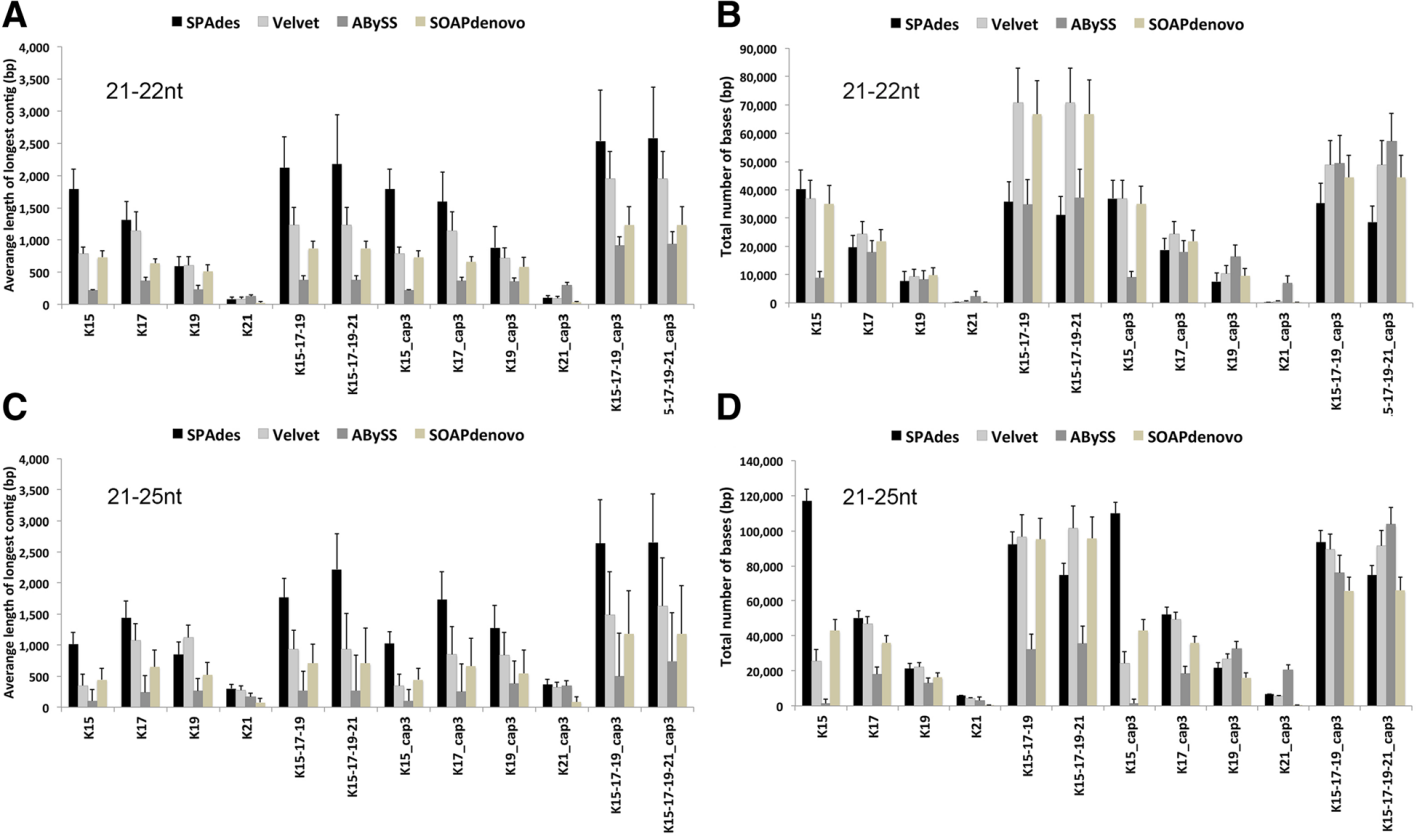
Comparison of SPAdes, Velvet, ABySS and SOAPdenovo assemblies using various kmer settings prior and after CAP3 scaffolding. Twelve samples were selected for the comparisons (see Additional file 1 for details). **a** Average longest assembled contigs for each kmer setting using 21–22 nt reads are shown. See text for kmer settings used. Assemblies after CAP3 scaffolding are denoted with ‘_cap3’. **b** Average total number of assembled bases for each kmer setting using 21–22 nt reads is shown. **c** Average longest assembled contigs for each kmer setting using 21–25 nt reads are shown. **d** Average total number of assembled bases for each kmer setting using 21–25 nt reads is shown

Preliminary de novo assembly of 12 quarantine samples sequenced in this study (Additional file 1) were evaluated with Velvet [16], SPAdes [17], ABySS [18] and SOAPdenovo [19] assemblers using individual kmer lengths of 15 (K15), 17 (K17), 19 (K19) and 21 (K21) as well as kmer sets of 15, 17, 19 (K15-17-19) and 15, 17, 19, 21 (K15-17-19-21). We found that SPAdes K15-17-19 and SPAdes K15-17-19-21 coupled with CAP3 [20] scaffolding produced the longest assembled sequences (Fig. 2a and c). Furthermore, we found that SPAdes K15-17-19 yielded a larger number of assembled bases than SPAdes 15-17-19-21 (Fig. 2b and d). SPAdes uses a unique approach to progressively build the assembled contigs using first the shortest kmer size in the first round, and then it builds upon the results of the previous round to continue to assemble sequences using additional user-defined longer kmer sizes (Additional file 3). This feature is not available to Velvet, ABySS and SOAPdenovo yielding a large fraction of redundant contigs assembled by individual kmers sizes that account for the increased total number of assembled bases prior and after CAP3 scaffolding (Fig. 2b and d), respectively. Overall we found that SPAdes K15-17-19 coupled with cap3 scaffolding produced the best results as compared to other tools and kmer settings tested (Fig. 2; Additional files 3 and 4).

### De novo assembly of viral genomes using small RNA subsets

**Fig. 3:**
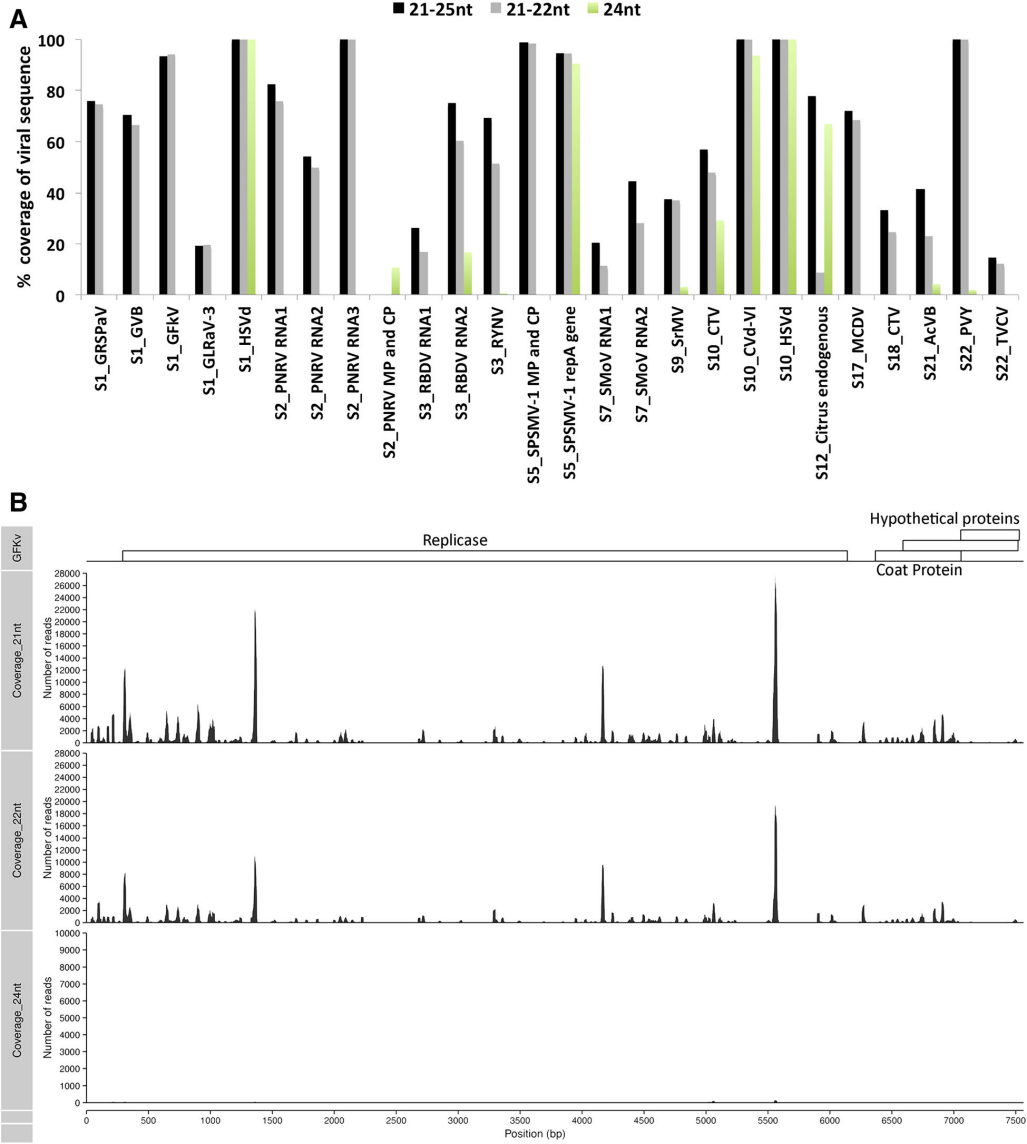
Comparison of viral genome coverage using subsets of small RNA reads. **a** Percentage coverage of viral genomes by contigs assembled using 21–25 nt, 21–22 nt or 24 nt small RNAs. S1 = *Vitis Vinifera*; S2 = *Prunus persica*; S3 = *Rubus idaeus*; S9 = *Miscanthus sinensis*; S10 = *Citrus medica*; S12 = *Citrus sp.*; S17 = *Pennisetum advena*; S21 = *Actinidia deliciosa*; S22 = *Nicotiana tabacum*; GRSPaV = Grapevine rupestris stem pitting-associated virus; GVB = Grapevine virus B; GFkV = Grapevine fleck virus; GLRaV-3 = Grapevine leafroll-associated virus; HSVd = Hop stunt viroid; PNRSV = prunus necrotic ringspot virus; RBDV = Raspberry bushy dwarf virus; RYNV = Rubus yellow net virus; SPSMV-1 = Sweetpotato symptomless mastrevirus 1; SMoV = Strawberry mottle virus; SrMV = Sorghum moasaic virus; CTV = Citrus tristeza virus; CVd-VI = Citrus viroid VI; MCDV = Maize chlorotic dwarf virus; AcVB = Actinidia virus B; CTV = TVCV = Tobacco vein-clearing virus. **b** Mapping of high quality reads with no mismatches onto a reference grapevine fleck virus (GVFv)

Typically de novo assembly of viral sequences has been conducted using 21 to 25 nt long reads. In this study, we compared the de novo assembly outcomes of viral sequences using three subsets of small RNA reads: i) 21–25 nt, ii) 21–22 nt, and iii) 24 nt long reads. We identified contigs with sequence similarity to known viral genomes in 12 samples (Fig. 3a, Table 1 and Additional file 5). In agreement with the known plant antiviral response activities of Dicer4 and Dicer2 enzymes [31, 34], most of the identified viral sequences were assembled using 21–22 nt reads (Fig. 3a and b). Only a Citrus endogenous pararetrovirus was more effectively assembled using 24 nt long reads than with 21–22 nt reads (Fig. 3a). Endogenous pararetrovirus sequences (EPRV) belonging to the plant virus family Caulimoviridae have been discovered in the genomes of a wide range of angiosperms preferentially integrated into AT dinucleotide repeats [35, 36]. Transcription of EPRV sequences along with flanking repeats may trigger a Dicer3-mediated silencing pathway that cleaves repeats and heterochromatin sequences into 24 nt long small interference RNAs [32, 37]. Users are encouraged to run in parallel 21–22 nt and 24 nt assembly workflows to screen for distinct types of viruses.

**Table 1:** Details of samples and the viruses and viroids detected using the ‘detect viruses and viroids’ workflow using 21–25 nt and 21–22 nt length reads. Additional information can be found on Additional file 6

		21–25 nt			21–22 nt			24 nt		
Host	Viruses detected	Number of contig hits	% Identity	% Coverage of viral genome by contigs	Number of contig hits	% Identity	% Coverage of viral genome by contigs	Number of contig hits	% Identity	% Coverage of viral genome by contigs
*Vitis vinifera* (S1)	GRSPaV	22	94.03	76.07	21	93.57	74.65	n.a.	n.a.	n.a.
	GVB^a^	13	96.82	70.5	13	97.23	66.54	n.a.	n.a.	n.a.
	GFkV	22	92.37	93.2	21	92.12	94.13	n.a.	n.a.	n.a.
	GLRaV-3	16	99.7	19.16	14	99.71	19.44	n.a.	n.a.	n.a.
	HSVd	2	100	100	1	100	100	2	100	100
*Prunus persica* (S2)	PNRSV RNA1^b^	9	98.6	82.63	8	98.68	75.76	n.a.	n.a.	n.a.
	PNRSV RNA2^b^	6	98.63	54.26	5	98.74	49.86	n.a.	n.a.	n.a.
	PNRSV RNA3^b^	2	98.85	100	1	98.66	100	n.a.	n.a.	n.a.
*Rubus idaeus* (S3)	RBDV RNA1 ^c^	5	97.83	26.41	4	97.89	16.98	n.a.	n.a.	n.a.
	RBDV RNA2 ^c^	8	98.77	75.08	6	98.68	60.51	3	99.15	16.76
	RYNV	19	98.08	69.14	15	97.23	51.49	1	98.08	0.66
*Brassica sp.* (S4)	PCV (RdRp)	2	52.12	62.14	2	69.38	41.72	n.a.	n.a.	n.a.
	ACV (RT)	2	67.5	57.44	2	52.92	61.08	n.a.	n.a.	n.a.
*Ipomoea batatas* (S5)	SPSMV-1 MP and CP	1	99.89	98.81	1	100	98.62	n.a.	n.a.	n.a.
*Fragaria ananassa* (S7)	SMoV RNA1	6	86.67	20.57	4	85.42	11.3	n.a.	n.a.	n.a.
	SMoV RNA2	11	87.59	44.62	6	87.13	28.26	n.a.	n.a.	n.a.
*Miscanthus sinensis (S9)*	SrMV	8	83.43	37.53	7	81.67	37.04	3	90.03	3.16
*Citrus medica* (S10)	CTV^d^	26	95.7	56.79	31	97.42	47.8	21	97.96	29.24
	CVd-VI^d^	2	99.41	100	2	99.42	100	1	99.36	93.69
	HSVd	2	99.09	100	2	99.09	100	2	98.84	100
*Citrus sp.* (S12)	Citrus endogenous pararetrovirus	42	90.26	77.92	3	91.35	8.87	31	89.07	66.94
*Pennisetum advena* (S17)	MCDV	29	98.45	72.14	27	98.81	68.35	n.a.	n.a.	n.a.
*Citrus latifolia* (S18)	CTV	30	98.67	33.03	21	98.69	24.75	n.a.	n.a.	n.a.
*Actinidia* (S21)	AcVB	33	93.05	41.25	18	94.24	23.21	4	91.75	4.22
*Nicotiana tabacum*	PVY	3	98.63	100	1	98.65	100	1	98.94	1.94
(S22)	TVCV	5	91.81	14.54	4	89.23	12.23	n.a.	n.a.	n.a.

Virus name: *GRSPaV Grapevine rupestris stem pitting-associated virus*, *GVB Grapevine virus B*, *GFkV Grapevine fleck virus*, *GLRaV-3 Grapevine leafroll-associated virus*, *HSVd Hop stunt viroid*, *PNRSV Prunus necrotic ringspot virus*, *RBDV Raspberry bushy dwarf virus*, *RYNV Rubus yellow net virus*, *PCV (RdRp) Persimmon cryptic virus* (RNA dependent RNA polymerase), *ACV (RT) Arisotelia chilensis virus* 1 (Reverse transcriptase), *SPSMV-1 Sweetpotato symptomless mastrevirus* 1, *SMoV* Strawberry mottle virus, *SrMV* Sorghum mosaic virus, *CTV* Citrus tristeza virus, *CVd-VI* Citrus viroid VI, *MCDV* Maize chlorotic dwarf virus, *AcVB* Actinidia virus B, *TVCV* Tobacco vein-clearing virus, *PVY* Potato virus Y

^a^Positive control for GVB identified using biological indexing at Post Entry Quarantine (PEQ)

^b^PEQ positive control for PNRSV detected using ELISA (Agdia)

^c^PEQ positive control for RBDV identified using ELISA (Agdia)

^d^PEQ positive control for CTV and CVd-VI detected using PCR-based assays

To assign sequence similarity of de novo assembled contigs to plant, viruses and viroid sequences using either BLASTN or BLASTX against NT and NR databases, respectively, a threshold of e-value 1e^−10^ is applied. The mean average alignment length for BLASTN and BLASTX hits assembled using the 21–22 nt pipeline was 256.85 bp and 327.32 bp, respectively (Additional file 5A and B). The minimal length of de novo assembled contigs that are compared against public databases is 40 bp. We found that for BLASTN alignment lengths of less than 50 bp the average nucleotide sequence identity was 98.32% ranging from 93.48% to up to 100% (Additional file 5A). In contrast, we identified two contigs with the lowest nucleotide sequence identities of 78.39% and 79.21% that had an alignment length of 944 bp and 178 bp, respectively (Additional file 5A). Similar results were observed for de novo assembled contigs produced using either the 21–25 nt (Additional file 5C) or 24 nt (Additional file 5E) pipelines. Our approach identifies viral sequences with high nucleotide sequence similarity to known viruses deposited in the NT database.

To identify more divergent viral sequences the BLASTX similarity to the NR database is used. We identified that the minimal protein alignment length reported for an e-value of 1e^−10^ was 28 amino acids corresponding to 84 bp. Inspection of protein alignment shorter than 50 amino acids for de novo assembled contigs produced using the 21–22 nt pipeline showed that the average amino acid sequence identity was 94.71% ranging from 61.90% to 100% (Additional file 5B). Similar BLASTX results were observed for contigs assembled using the 21–25 nt pipeline (Additional file 5D). Although most samples processed by the 24 nt pipeline also showed similar BLASTX results, we identified a *Pennisetum advena* (S17) and a *Citrus latifolia* (S18) that did not yield a hit against viral proteins (Additional file 5F).

Most viral sequences found in this study have sequence similarity to ssRNA (+) viruses (Additional file 6). To evaluate the length distribution of small RNAs making up the viral sequences we mapped quality trimmed reads onto a reference *Grapevine fleck virus* (GFkV) genome, and showed that out of 376,891 mapped reads with zero mismatches 61.93%, 37.86% and 0.21% had lengths of 21 nt, 22 nt and 24 nt, respectively (Fig. 3b). We also aligned reads onto a *Citrus tristeza virus* (CTV) genome, yielding 3.8 million mapped reads with no mismatches, of which 52.3%, 45.74% and 1.95% were 21 nt, 22 nt and 24 nt in length, respectively (Additional file 7A). These findings correlate with the notion that Dicer4 is the main antiviral response enzyme in leaf tissues [31].

We detected three viroids including *Hop stunt viroid* (HSVd) and Citrus viroid VI (CVd-VI) in citrus, and HSVd in grapevine (Fig. 3a and Additional file 6). Interestingly, all three viroid sequences were similarly assembled using either 21–22 nt or 24 nt small RNA subsets. Mapping of high quality trimmed reads with no mismatches onto the reference HSVd genome (KT725429) yielded 483,523 mapped reads, of these 48.52%, 12.70% and 38.78% had a length of 21 nt, 22 nt and 24 nt, respectively (Additional file 7B). The identified viroids in this study are predicted to form a hairpin-like RNA secondary structure that may be recognised not only by the antiviral response pathway but also by other plant small RNA pathways (Additional file 8). The possible intervention of two or more plant small RNA pathways may result in the cleavage of viroids into 21 nt, 22 nt and 24 nt long sequences. Furthermore, to identify new to science viroids the complete assembly of their genome using 21–22 nt or 24 nt small RNA subsets can be applied as filtering criteria.

To evaluate the fraction of small RNA reads making up de novo assembled sequences we mapped high quality adaptor clipped reads onto assembled contigs using Bowtie [38] with up to 3 mismatches. We found on average that 61.98% of the reads were not mapped onto assembled contigs (range from 19.25% to up to 88.40%) (Additional file 9). Inspection of the mapped reads, showed that on average 20.53%, 11.17%, 6.16% and 0.16% of these reads were anchored onto assembled contigs with sequence similarity to plants, viruses, sequences with no similarity to plants or viral sequences (unknown) and viroids, respectively (Additional file 9). The fraction of reads anchored onto viral sequences ranged from 0.09% to up to 75.74%.

We then aimed to define the minimum amount of small RNA data that should be collected for the surveillance and diagnosis of viral sequences. As a case study we used a *Prunus persica* (S2) sample and tested the diagnosis of the tripartite *Prunus necrotic ringspot virus* (PNRSV) using subsets of 1 M, 2 M, 5 M, and 10 M high quality small RNA reads. We found that when using 10 M reads for de novo assembly we detected 37.83%, 21.66% and 88.63% of the PNRSV RNA1, RNA2 and RNA3, respectively (Additional file 10). The use of smaller subsets of reads resulted in limited assembly of PNRSV RNA1 and RNA2 sequences. To increase the confidence in the detected viral sequences we propose to use at least 10 M small RNA reads per quarantined sample. It is critical to detect exotic viral sequences in imported quarantined plant samples to protect domestic plant industries, native plant biodiversity and prevent economic and social impacts to the broader community.

### Assembly issues using combined 21–25 nt small RNAs

**Fig. 4:**
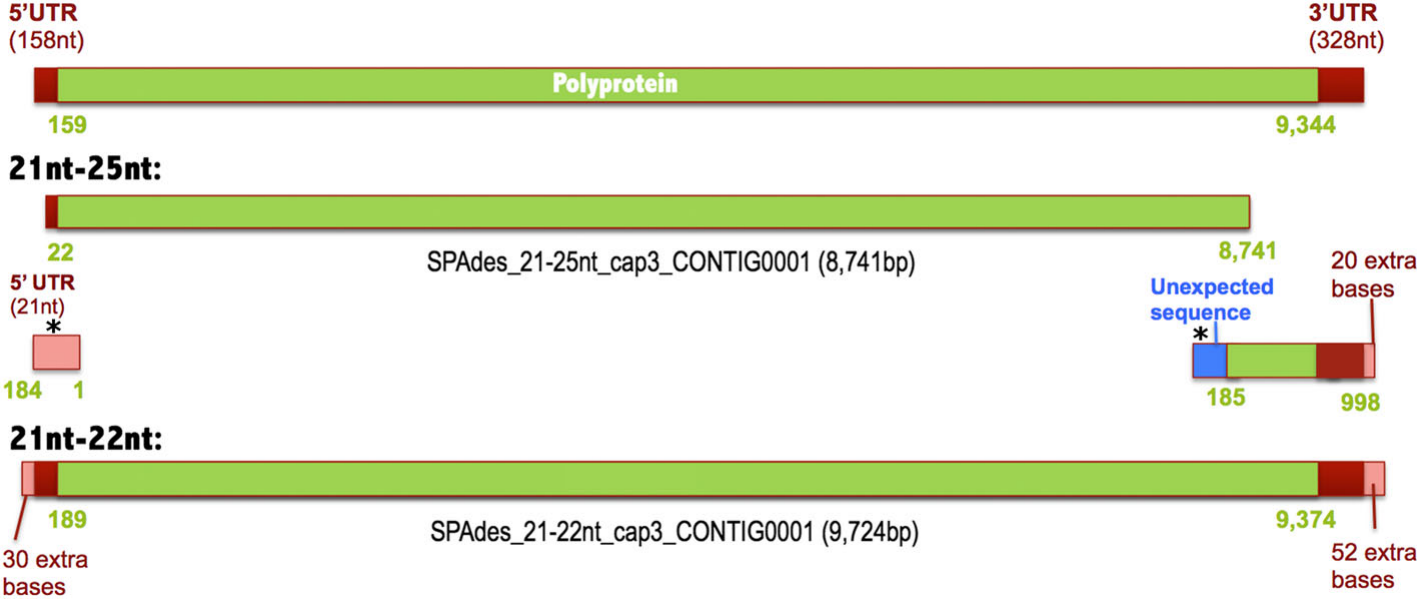
Assembly of a Potato virus Y genome using 21–25 nt and 21–22 nt reads

Collected small RNAs from plant host samples contain the products of several biological pathways including antiviral response, heterochromatin and transposon silencing, and microRNA biogenesis. We found that conducting de novo assembly using 21–25 nt small RNA sequences resulted in a partial assembly of a *Potato virus Y* (PVY) genome (Fig. 4). Two contigs were assembled, one encoding most of the polyprotein region and another encoding part of the 3′end region of the genome. The shorter contig included 184 bp at the 5′-end with no sequence similarity to the terminal region of the PVY polyprotein, instead reverse complementation of these 184 bp produced a match to the beginning of the PVY genome. In contrast to these findings, de novo assembly using only a 21–22 nt subset of small RNAs resulted in the complete assembly of the PVY sequence including 30 and 52 extra bases in their 5′-end and 3′-end terminal regions, respectively (Fig. 4). These findings suggest that improved and accurate assembly results are obtained using specific 21–22 nt small RNAs produced by the plant antiviral response pathway [31, 34].

### Comparison of toolkit outcomes with VirFind

We subjected 18 raw RNA-Seq datasets to evaluate the performance of VSD toolkit as compared to VirFind [11]. Summary of results with the VirFind pipeline are outlined in Additional file 11. VirFind identified viral sequences in all 18 samples, of these in 13 samples the VSD toolkit found the same viral sequences (Additional file 11A), while 5 samples showed viral hits unique to the VirFind pipeline (Additional file 11B). Inspection of the later samples showed hits to viral sequences from plants, algae, fungi, invertebrate and vertebrates hosts (Additional files 11B and 12). We aligned the adaptor clipped reads onto the assembled VirFind contigs for these samples and found reads from 19 nt to up to 29 nt aligned onto contigs for these samples highlighting the broad spectrum of reads that are used in the VirFind pipeline (Additional file 13). To test if these unique hits in the VirFind pipeline may potentially be chimeric sequences with host plant sequences, we conducted a BLASTX screening against a combined plant and virus protein database and reported the top hit. Additional file 14 shows that 66.7% of the VirFind contigs annotated as viral sequences have a top hit to a plant or an insect virus. These findings highlight further the susceptibility to assemble chimeric sequences and/or plant sequences with similarity to viruses (i.e. RNA dependent RNA polymerases) when using a broad spectrum of small RNA read lengths collected from plant hosts.

Comparison of the viral sequences commonly assembled by VirFind and our approach showed that the VSD toolkit assembled an average of 29.31% and 21.56% more of the 25 viral sequences found in 12 plant samples using the 21–25 nt and 21–22 nt workflows, respectively (Additional file 15). The largest increases in the assembly of the known viral sequences were for the *Citrus endogenous pararetrovirus* (74.29%), *Grapevine freck virus* (66.98%), *Potato Virus Y* (63.93%) and *Prunus necrotic ringspot virus* RNA1 (50.82%). Only in the case of the *Grapevine leafroll-associated virus 3* (GLRaV-3) did VirFind assemble a greater (28.48%) proportion of the sequences than the VSD toolkit (19.44%) (Additional file 15).

Both the VSD toolkit and VirFind detected similarly all three viroids sequences (Additional file 12). In the case of the *Citrus medica* Endogenous Pararetrovirus (CmeEPRV) the VSD toolkit assembled 77.92%, 8.87% and 66.94% of the genome using 21–25 nt, 21–22 nt and 24 nt assembly workflows, respectively. In contrast, VirFind only assembled 3.63% of the CmeEPRV genome sequence (Additional file 15).

Overall we show that VSD toolkit produced improved and more accurate results than VirFind. It is critical to utilise the specific by-products of the plant immune defence pathway against viruses and viroids to assemble highly accurate viral sequences that reflect the active host antiviral response. Detection of exotic viral pathogens at the border is critical to safeguard plant industries and their access to national and international trade markets.

## Conclusions

We have implemented an automated viral surveillance and diagnosis toolkit using the Yabi web-based analytical environment that enables improved detection of viruses and viroids pathogens. We found that all single stranded RNA (+) viruses found in this study were assembled using exclusively 21–22 nt small RNAs, while viroids were equally assembled using 21–22 nt or 24 nt subsets. The use of specific small RNA subsets increases specificity of the identified viral sequences as it reflects the antiviral response activity of the plant hosts.

## Additional files

Additional file 1: Adaptor clipped and quality trimmed read statistics of small RNA-Seq datasets. Clean reads = Adaptor clipped small RNA reads provided by BGI service provider; QC = Quality Control consisting of adaptor clipping and poor base call trimming; n.a. = not available. (XLSX 44 kb)
Additional file 2: Yabi Viral Surveillance and Diagnosis (VSD) toolkit User-guide. (PDF 3156 kb)
Additional file 3: Boxplot comparison of SPAdes assemblies using individual kmers, sets of kmers and CAP3 scaffolding. (JPG 527 kb)
Additional file 4: Paired sample t-test of contigs produced by SPAdes, Velvet, ABySS and SOAPdenovo assemblers. Our optimised ‘SPAdes K15-17-19_cap3’ (SPAdes assembly; dataset = 21–22 nt; kmer set =15,17 and 19; and scaffolded with CAP3) (SP22_K15-17-19_cap3) assembly was compared against all other kmer settings and/or tools. A) SPAdes - Longest assembled contigs (21–22 nt pipeline); B) SPAdes - Total number of assembled bases for contigs > =100 nt (21–22 nt pipeline); C) SPAdes - Longest assembled contigs (21–25 nt pipeline); D) SPAdes - Total number of assembled bases for contigs > =100 nt (21–25 nt pipeline); E) Comparison of longest assembled contig using Velvet against SP22_K15-17-19_cap3; F) Comparison of longest assembled contig using ABySS against SP22_K15-17-19_cap3; G) Comparison of longest assembled contig using SOAPdenovo against SP22_K15-17-19_cap3. (XLSX 19 kb)
Additional file 5: BLASTN and BLASTX sequence similarity statistics for de novo assembled contigs using the 21–22 nt (A and B), 21–25 nt (C and D) and 24 nt (E and F) pipelines, respectively. Twelve small RNA-Seq samples generated in this study with nucleotide similarity to viral pathogens were compared. See Additional file 1 for details of the selected samples. (JPG 809 kb)
Additional file 6: Statistics of the viral sequences assembled using 21–25 nt, 21–22 nt and 24 nt small RNA subsets. *Virus name: GRSPaV = *Grapevine rupestris stem pitting-associated virus*; GVB = *Grapevine virus B*; GFkV = *Grapevine fleck virus*; GLRaV-3 = *Grapevine leafroll-associated virus*; HSVd = *Hop stunt viroid*; PNRSV = *Prunus necrotic ringspot virus*; RBDV = *Raspberry bushy dwarf virus*; RYNV = *Rubus yellow net virus*; SPSMV-1 = *Sweetpotato symptomless mastrevirus 1*; SMoV = *Strawberry mottle virus*; MsiMV = *Miscanthus sinensis mosaic virus*; CTV = *Citrus tristeza virus*; CVd-VI = *Citrus viroid VI*; MCDV = *Maize chlorotic dwarf virus*; AcVB = *Actinidia virus B*; TVCV = *Tobacco vein-clearing virus*. **Virus type: RT = retro-transcribing virus; ssRNA + = ssRNA positive-strand virus; ssDNA = single-stranded DNA virus; dsRNA = double-strand RNA virus; V = viroid. (XLSX 49 kb)
Additional file 7: Mapping of high quality adaptor-clipped and quality trimmed small RNAs with no mismatches onto the reference genomes: A) Citrus tristesa virus (CTV; accession number AB046398). Domains of the CTV genomes are denoted. B) Hop Stunt Viroid (HSVd). (JPG 702 kb)
Additional file 8: Predicted RNA secondary structure of viroids found in this study. Minimal free energy RNA secondary structure encoding base-pair probabilities are shown for S1_HSVd (sample 1 – Hop Stunt Viroid), S10_CVD-VI (sample 10 – Citrus Viroid VI), and S10_HSVd (sample 10 – Hop Stunt Viroid). (JPG 1406 kb)
Additional file 9: Distribution of mapped and unmapped small RNA reads for quarantined samples generated in this study. A) Percentage of unmapped and mapped reads onto de novo assembled contigs with sequence similarity to plants; viruses, viroids and unknown are shown. B) Distribution of the percentage of mapped reads for each sample along with the total number of distinct viral sequences (viruses/viroids) are shown. Details for each sample can be found in Additional file 1. (JPG 477 kb)
Additional file 10: De novo assembly of PNRSV RNA1, RNA2 and RNA3 viral sequences using five randomly generated subsets of 1 M, 2 M, 5 M, 10 M and all adaptor clipped small RNA reads. (PDF 5 kb)
Additional file 11: Summary of VirFind screening of small RNA-Seq datasets collected from quarantined plants. A) VirFind blastn results. Identified viruses/viroids were also identified using our VSD toolkit. B) VirFind blastx results. Identified viruses/viroids were only found using the VirFind pipeline. Hit to viruses from vertebrates, invertebrates, fungi or algae are shown in red background. (XLSX 38 kb)
Additional file 12: VirFind BLASTX results for contigs with sequence simalrity to viruses or viroids. (XLSX 50 kb)
Additional file 13: Mapping of adaptor clipped reads onto VirFind assembled contigs for samples S4, S11, S13, S14, S15 and S16. Details of samples can be found in Additional file 1. (PDF 5 kb)
Additional file 14: VirFind contigs annotated with hits to viruses were run through the Yabi VSD toolkit pipeline. Top BLASTX hits (Evalue = < 1e-5) to a combined plant and virus protein database are reported. VirFind assembled contigs with a top hit to a plant virus are highlighted in green background. (XLSX 59 kb)
Additional file 15: Comparison of genome coverage of known viral sequences by contigs assembled by the VSD toolkit and VirFind analysis pipelines. (JPG 1197 kb)

## Abbreviations

ABySS: A parallel assembler for short read sequence data
ACV (RT): *Arisotelia chilensis* virus 1 (Reverse transcriptase)
AcVB: Actinidia virus B
AWS: Amazon web services
BLAST: Basic local alignment search tool
ConDeTri: A content dependent read trimmer for illumina data
CTV: *Citrus tristeza* virus
CVd-VI: Citrus viroid VI
EC2: Elastic compute cloud
ELISA: Enzyme-linked immunosorbent assay
EPRV: Endogenous pararetrovirus sequences
GFkV: Grapevine fleck virus
GLRaV-3: Grapevine leafroll-associated virus
GRSPaV: Grapevine rupestris stem pitting-associated virus
GVB: Grapevine virus B
HSVd: Hop stunt viroid
K15: de novo assembly using kmer size of 15
K15-17-19: de novo assembly using a combined kmer set of 15, 17 and 19
K15-17-19-21: de novo assembly using a combined kmer set of 15, 17, 19 and 21
K17: de novo assembly using kmer size of 17
K19: de novo assembly using kmer size of 19
K21: de novo assembly using kmer size of 21
MCDV: Maize chlorotic dwarf virus
NGS: Next generation sequencing
PCR: Polymerase chain reaction
PCV (RdRp): Persimmon cryptic virus (RNA dependent RNA polymerase)
PEQ: Post entry quarantine
PNRSV: Prunus necrotic ringspot virus
PVY: Potato virus Y
RBDV: Raspberry bushy dwarf virus
RNA-Seq: RNA sequencing
RYNV: Rubus yellow net virus
SAM: Sequence alignment/map format
siRNA: Small interference small RNA
SMoV: Strawberry mottle virus
SOAPdenovo: Short oligonucleotide analysis package for short read de novo assembly
SPAdes: A genome assembly algorithm
SPSMV-1: Sweetpotato symptomless mastrevirus 1
SrMV: Sorghum mosaic virus
ssRNA: Single-strand RNA
TVCV: Tobacco vein-clearing virus
Velvet: Algorithm package for de novo genome assembly and short read alignment
VirFind: Virus find pipeline
viRNA: Virus-derived siRNA
VSD: Viral surveillance and diagnosis toolkit
Yabi: Yet another bioinformatics interface
