# Supplementary material for: An internet-based bioinformatics toolkit for plant biosecurity diagnosis and surveillance of viruses and viroids
Source: BMC Bioinformatics. 2017 Jan 11;18:26. doi: 10.1186/s12859-016-1428-4 (PMC5225587; doi:10.1186/s12859-016-1428-4)
Supplement: Additional file 2: — Yabi Viral Surveillance and Diagnosis (VSD) toolkit User-guide. (PDF 3156 kb) [file 12859_2016_1428_MOESM2_ESM.pdf]

Toolkit for Plant Biosecurity Diagnosis and Surveillance of Viruses and Viroids – USER GUIDE

Table of Contents

Background to Yabi ... 3

Log in ... 3

The Design Tab ... 3

The left panel, which displays the available tools ... 3

The middle panel, where you build a workflow ... 3

The right panel, where you select files or change parameters ... 3

Build a workflow part 1 ... 4

Build a workflow part 2 ... 5

Build a workflow part 3 ... 6

Select a file part 1 ... 7

Select a file, part 2 ... 8

Set tool parameters ... 9

The Jobs Tab ... 10

The left panel, which displays the list of jobs ... 10

The middle panel, which displays the tools used in the workflow ... 10

The right panel, which displays the output ... 10

Jobs running ... 11

File outputs part 1 ... 12

File outputs part 2 ... 13

The Files Tab ... 14

Transfer files ... 15

The Account Tab ... 16

Change password ... 17

The WorkFlows - Overview ... 18

Available pipelines ... 18

Virus and Viroid Detection and Detection of Novel Viroids ... 19

Mapping to Reference Genome ... 20

The WorkFlows – Tool Details ... 21

Virus and Viroid Detection and Detection of Novel Viroids ... 21

Tool 1: Select File ... 21

Tool 2: ConDeTri ... 21

Tool 3: Extract reads (21-25nt, 21-22nt, or 24nt) ... 21

Tool 4: Spades 3.5 Wrapper ... 21

Tool 5: CAP3 pipeline ... 22

Tool 6: Extract and rename contigs ... 22

Tool 7: blastn (virus, viroid and plant) ... 22

Tool 8: blastx (virus and plant) ... 23

Tool 9: Extract blast hits 23
Tool 10: Extract contigs- plant, virus, no hits 24
Tool 11: PLANT blastn output 24
Tool 12: VIRUS blastn output 24
Tool 13: PLANT blastx output 24
Tool 14: VIRUS blastx output 24
Tool 15: Filter contigs 200-460nt 24
Tool 16: Identify circular contigs 25
Mapping to Reference Genome 25
Tool 1: Select File 25
Tool 2: bowtie-build 25
Tool 3: bowtie 25
Tool 4: samtools view 25
Tool 5: samtools sort 26
Tools 6: samtools index 26

The WorkFlows – Output Files Details 27
Virus and Viroid Detection and Detection of Novel Viroids 27
Tool 1: Select File 27
Tool 2: ConDeTri 27
Tool 3: Extract reads (21-25nt, 21-22nt, or 24nt) 27
Tool 4: Spades 3.5 Wrapper 27
Tool 5: CAP3 pipeline 28
Tool 6: Extract and rename contigs 28
Tool 7: blastn (virus, viroid and plant) 28
Tool 8: blastx (virus and plant) 28
Tool 9: Extract blast hits 28
Tool 10: Extract contigs- plant, virus, no hits 28
Output tools: 29
Tool 11: PLANT blastn output 29
Tool 12: VIRUS blastn output 30
Tool 13: PLANT blastx output 30
Tool 14: VIRUS blastx output 30
Tool 15: Filter contigs 200-460nt 30
Tool 16: Identify circular contigs 30
Mapping to Reference Genome 30
Tool 1: Select File 30
Tool 2: bowtie-build 30
Tool 3: bowtie 31
Tool 4: samtools view 31
Tool 5: samtools sort 31
Tools 6: samtools index 31

References 31

Background to Yabi

Log in

Navigate to the site https://ccgapps.com.au/yabi/login/?next=/yabi/ and log in with your supplied username and password.

If you have forgotten your password, contact the Yabi team by email at: yabi@ccg.murdoch.edu.au

The Design Tab

The Design Tab is where you create workflows, or select saved workflows. The Design Tab has three panels:

The left panel, which displays the available tools

- You may search for tools in 'Find tool'
- Click 'show all' to show all tools
- 'Use selection to auto filter' may be turned on or off as desired
- Click on a tool to view a description, and the types of files it accepts and outputs.
- Press 'add' or drag and drop tools to the middle panel to build a workflow.
- Saved workflows are available at the bottom of this panel

The middle panel, where you build a workflow

- Tools or workflows added from the left panel appear here
- Drag and drop tools in the order required
- Click on each tool to select files or change parameters in the far right panel.
- The default options for each parameter automatically, but can be changed in most cases
- Use the 'Tags' feature to mark your workflows to enhance searching
- 'Save' the workflow to reuse it at a later date
- Name your workflow
- Click 'run' when you are ready to run your workflow
- Note that mandatory fields are marked red if they are not correctly filled. Your job will not be able to be submitted until mandatory fields are correctly filled.

The right panel, where you select files or change parameters

- Mandatory and common parameters appear first
- To see all available parameters, click 'show all options'

Build a workflow part 1

Workflows are built by dragging and dropping or adding the tools listed on the left into the workflow panel.

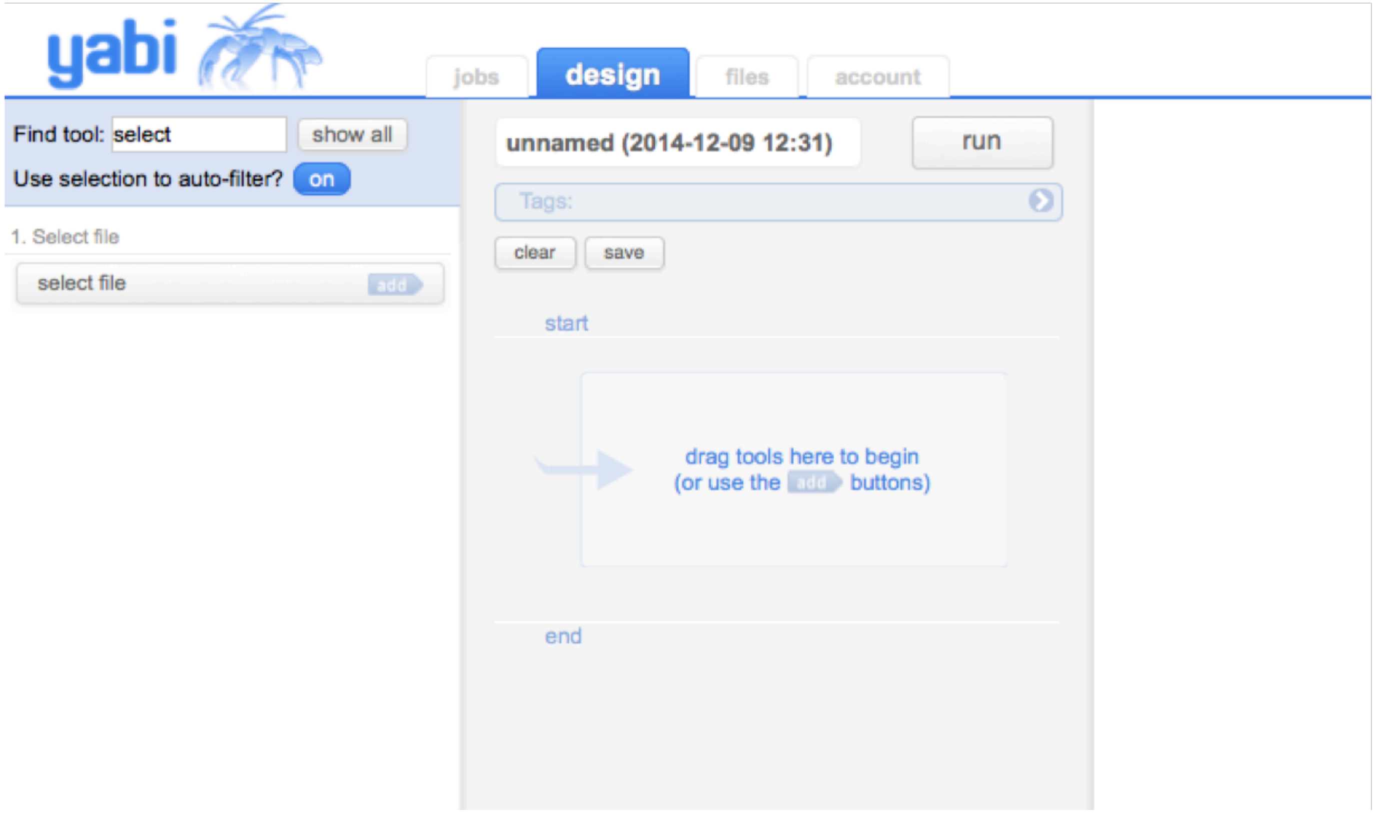

Build a workflow part 2

Click on ‘show all’ to show the list of all available tools, and turn ‘Use selection to auto-filter’ off to show all available tools.

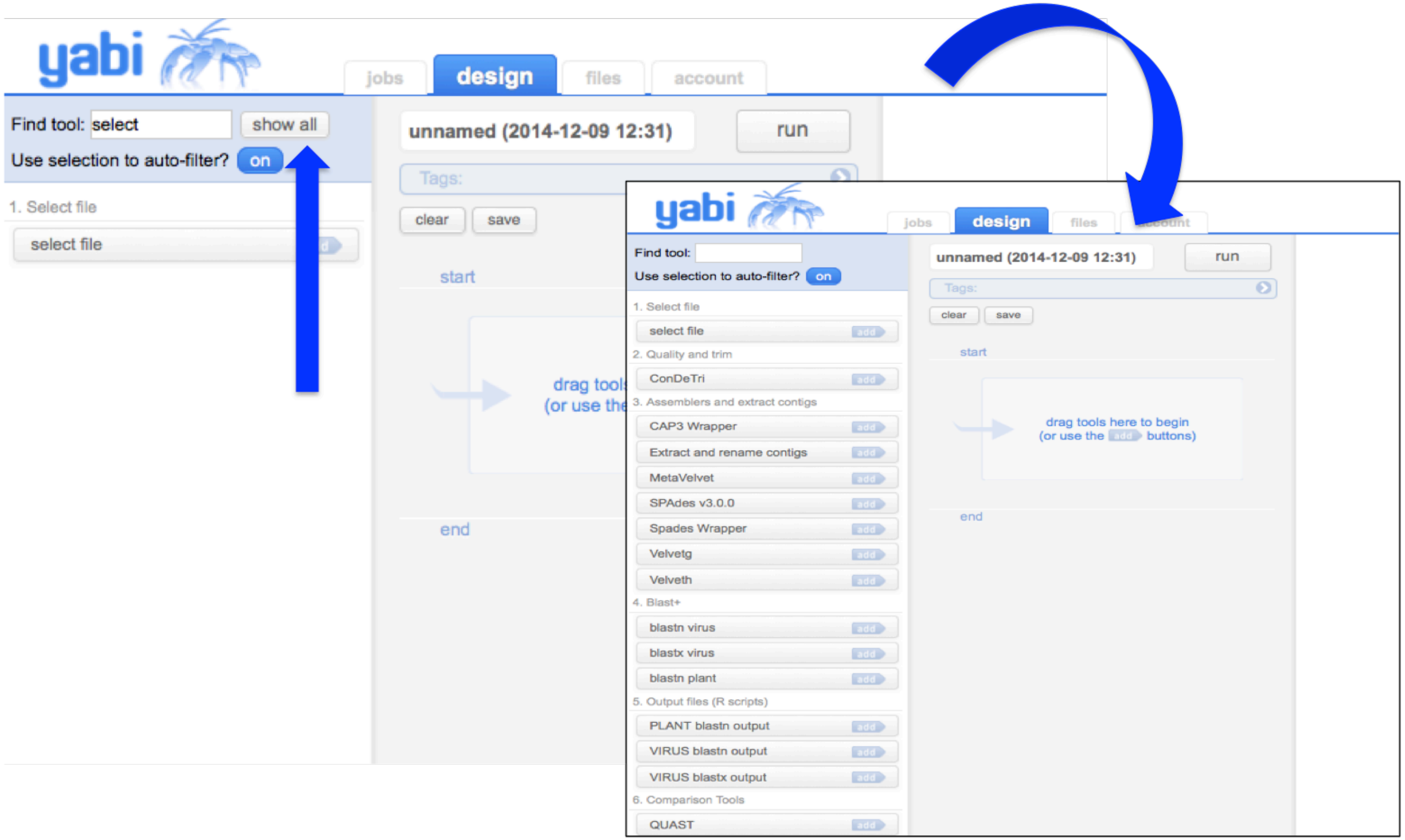

Build a workflow part 3

Add a tag to your workflow (optional) by clicking on the arrow to the right. Multiple tags should be separated by a comma. Name your workflow (optional).

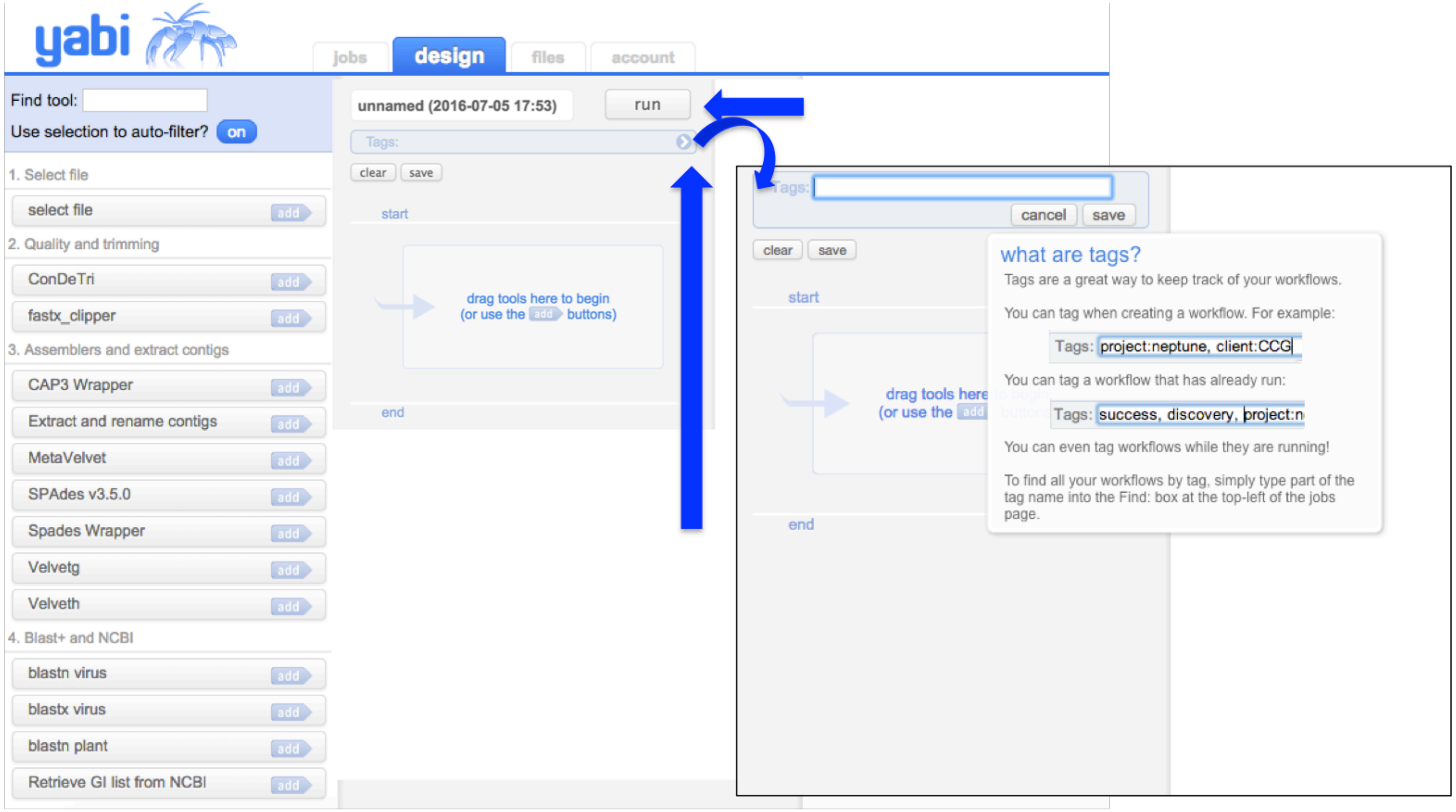

Select a file part 1

Clicking on the tool in the tool panel displays a description. Red boxes indicate mandatory options that need to be filled in (i.e. select a file). To select a file, first click on the 'Select file' Tool in the middle panel. Then, click on the folder of choice in the right panel. The arrow takes you back to the root file system.

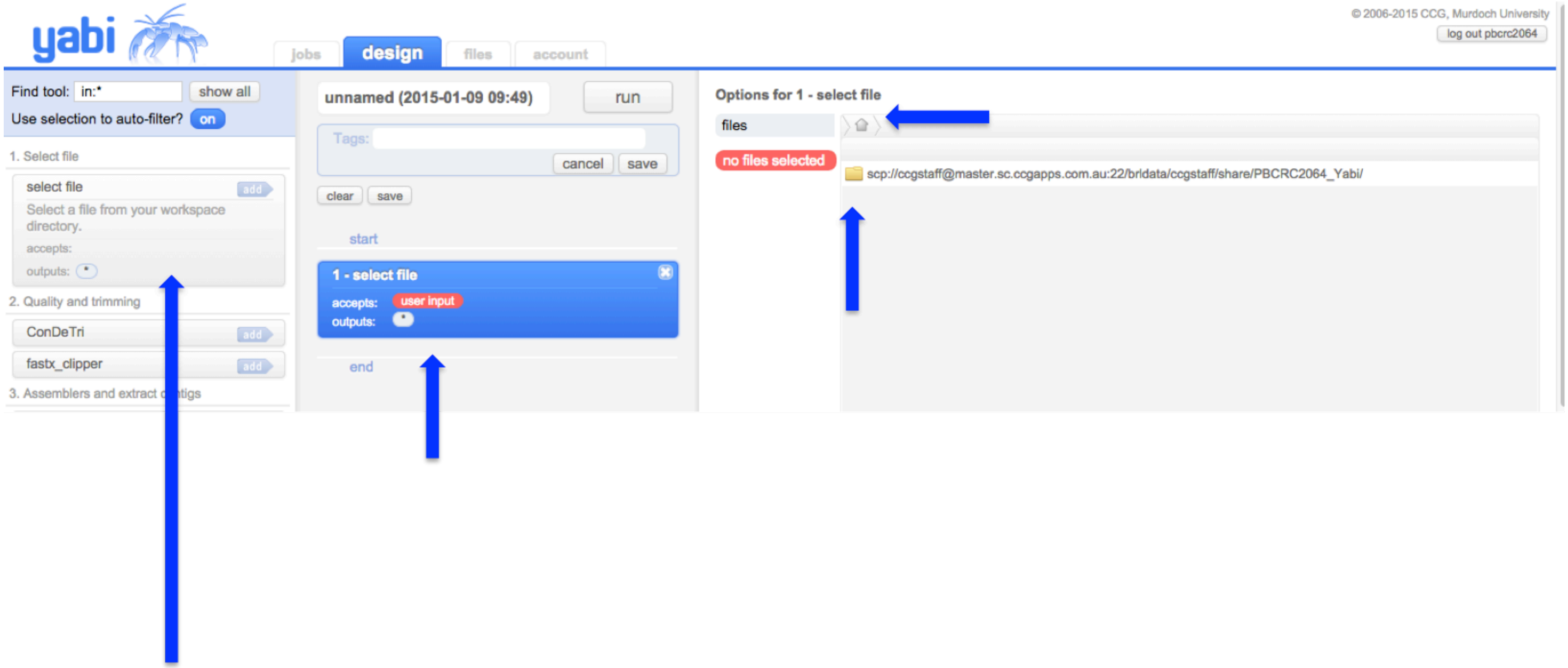

Select a file, part 2

Files can be previewed by clicking the magnifying glass icon. Files can also be uploaded from your desktop by clicking 'Select File' and 'Upload'. Multiple files can be selected.

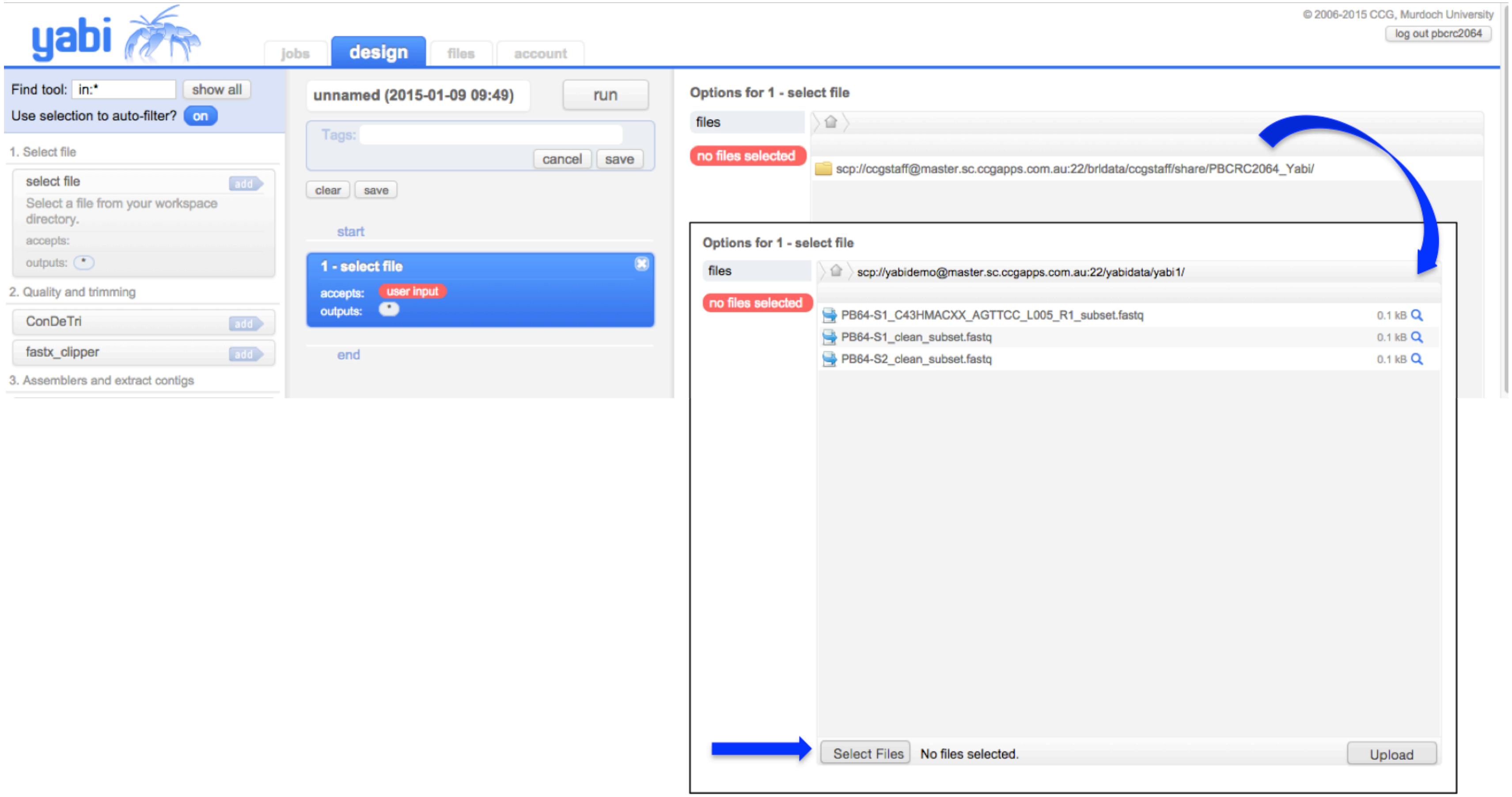

Set tool parameters

File types that are accepted by the tool are listed in the tool description and in the right panel. Click ‘Show all options’ to show all available tool parameters. Click on ‘run’ when you are ready to submit your workflow.

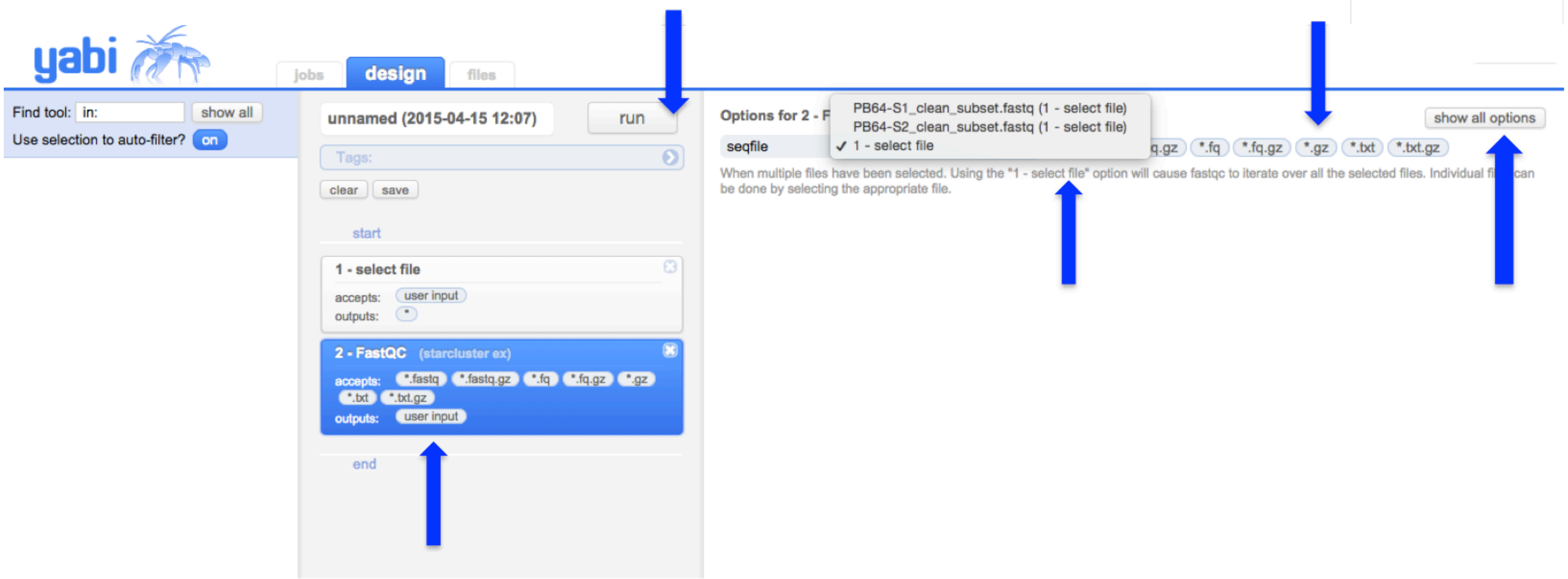

The Jobs Tab

The Jobs Tab is where you view the results of completed workflows, or t[illegible] of running workflows. The Jobs Tab has three panels:

The left panel, which displays the list of jobs

- All submitted workflows are displayed here
- A green tick means the workflow has successfully complet[illegible]
- A yellow exclamation point indicates an error has occurre[illegible] the workflow- this may simply mean more time is require[illegible] complete a step
- A red cross sign indicates the workflow was not able to co[illegible]
- A spinning wheel indicates a job is currently running.
- Search for jobs through the 'Find' text box, or utilise the da[illegible] and status buttons to search
- Click on the job to see the tools used in the workflow appe[illegible] middle panel

The middle panel, which displays the tools used in the workflow

- If a job is still running, the green bar indicates how far the [illegible] progressed
- The workflow may be re-used, saved, aborted, or shared

The right panel, which displays the output

- Output files for each tool can be previewed or downloaded
- The tool options used are saved and may also be viewed

Jobs running

Previously run jobs may be found through a search of job name, date range, or run status. Workflows may be reused, saved, aborted or shared with others. Green ticks indicate the job/ask has completed successfully, while the spinning wheel indicates the job/task is currently running.

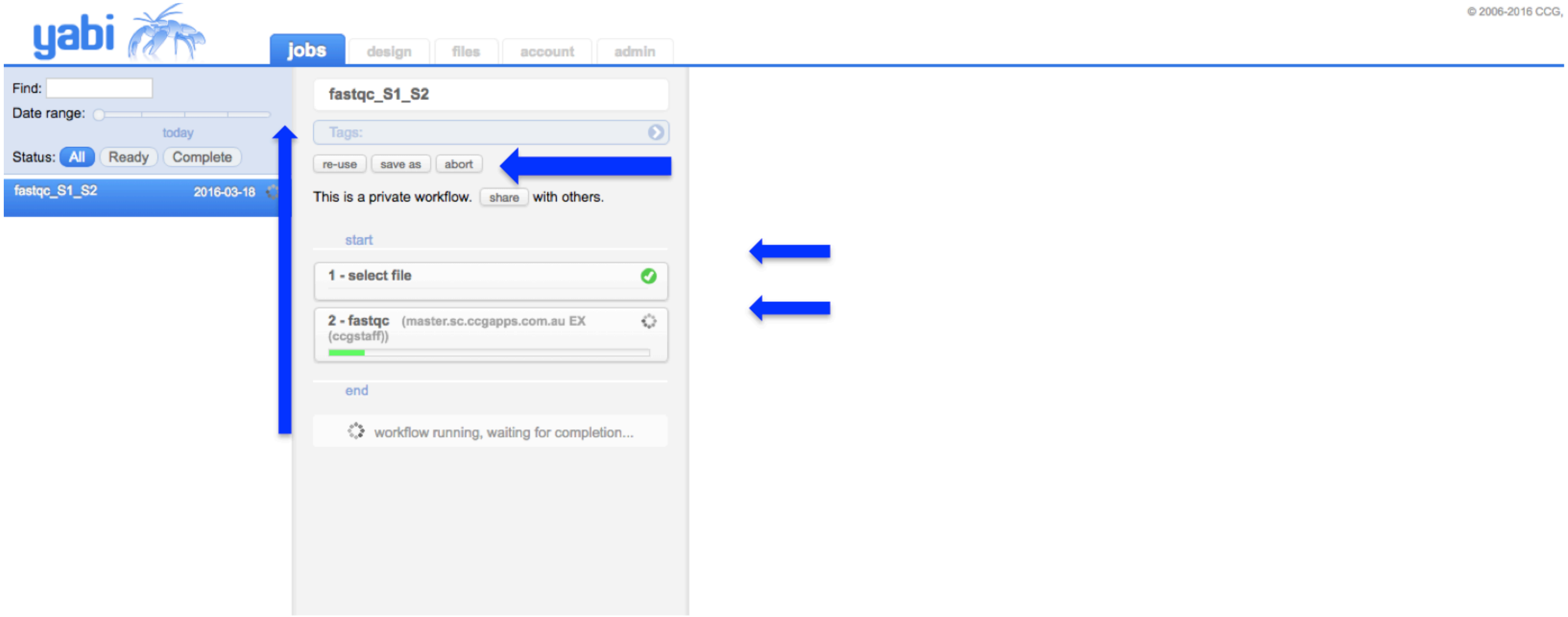

File outputs part 1

File outputs can be previewed by clicking the blue magnifying glass, or downloaded by clicking the green arrow.

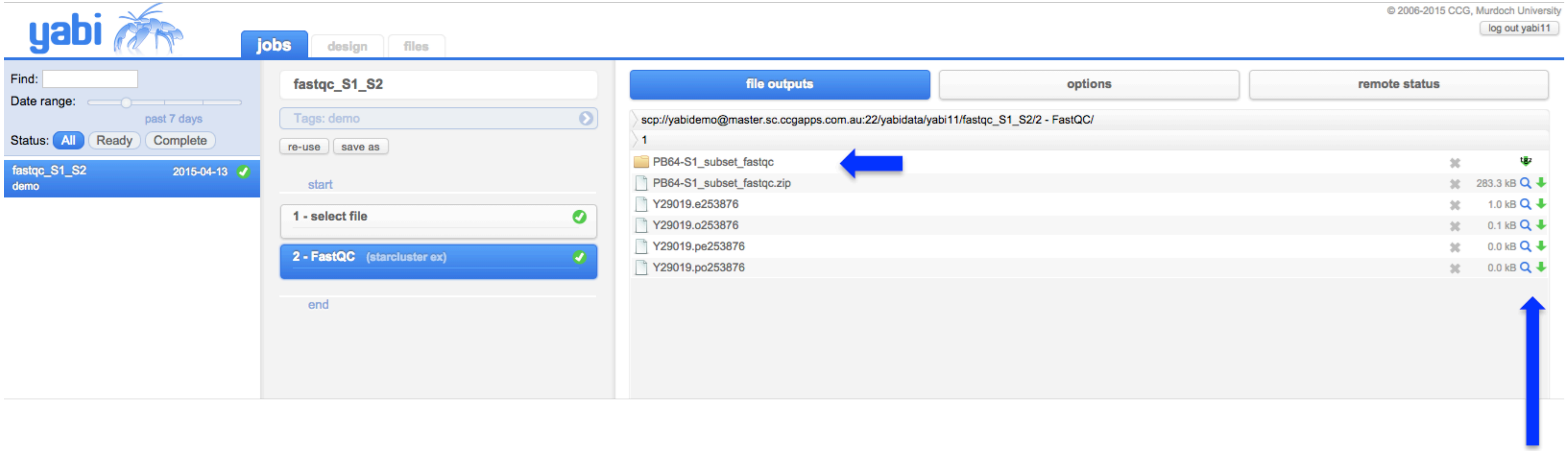

File outputs part 2

The tool options that were used are also captured, and may be viewed.

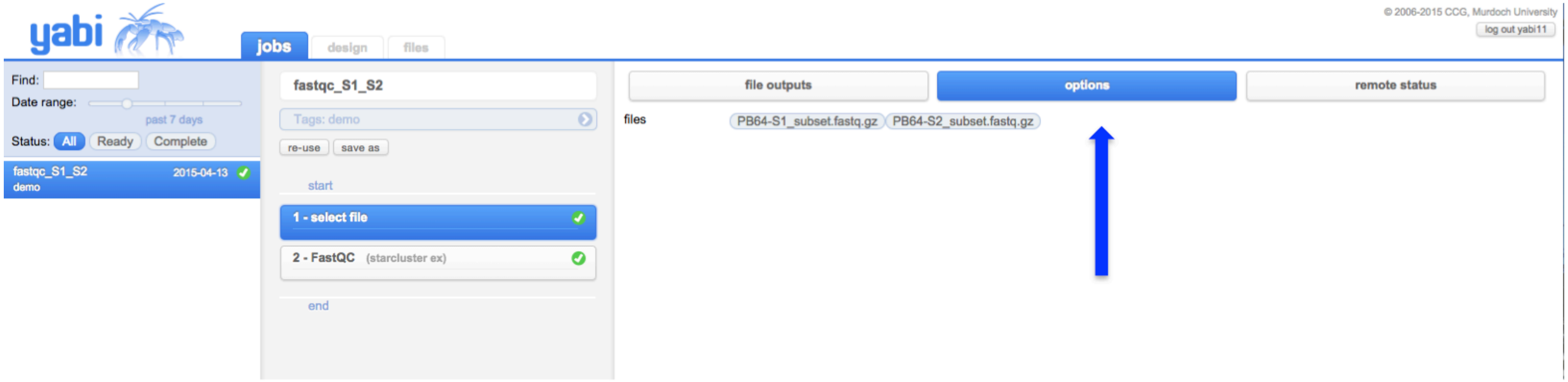

The Files Tab

The Files Tab is where you view your Backends (links to various high performance computing resources) and files. The split screen allows files [illegible] able to be dragged and dropped between different back ends. Files may [illegible] uploaded from your desktop.

Transfer files

To transfer files between directories or backends, drag and drop files from the right to the left, or left to the right panels. Files can be deleted by clicking the 'cross', previewed by clicking the 'blue magnifying lass' or downloaded by clicking the 'green arrow'.

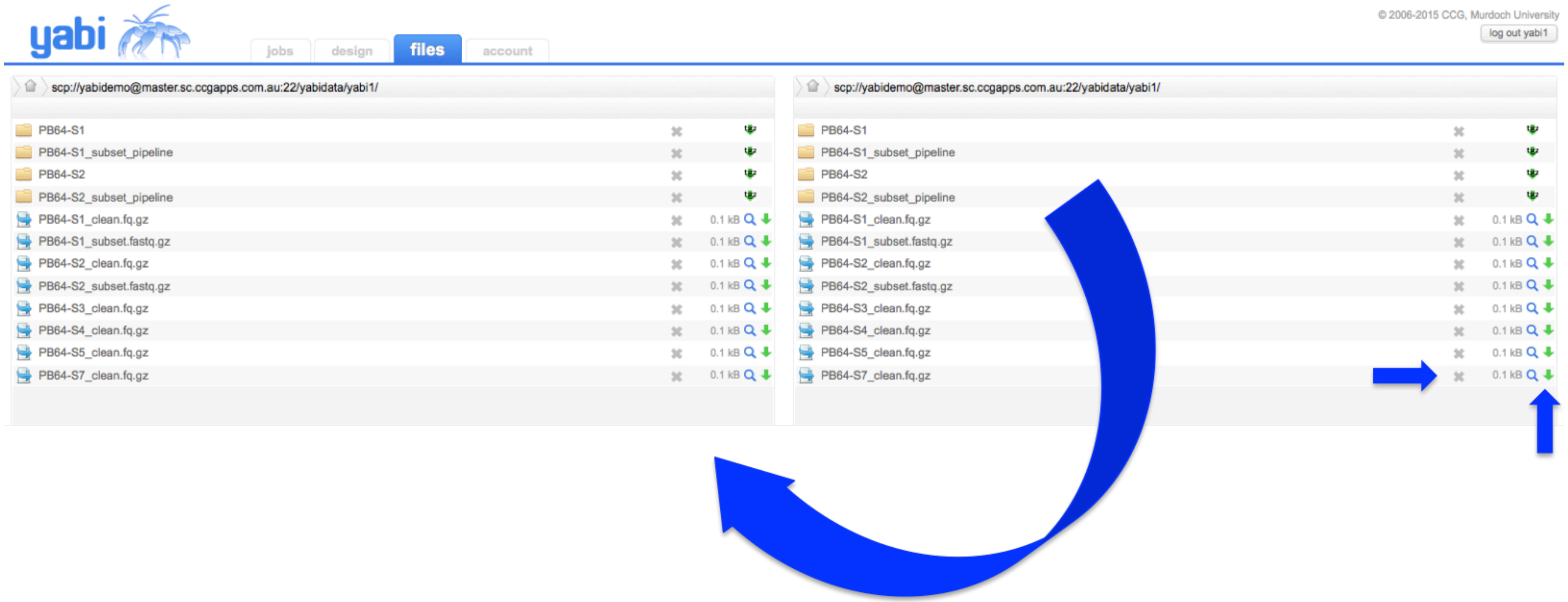

The Account Tab

The Account Tab is where you may change your password.

Change password

You may change your password by navigating to the Accounts Tab.

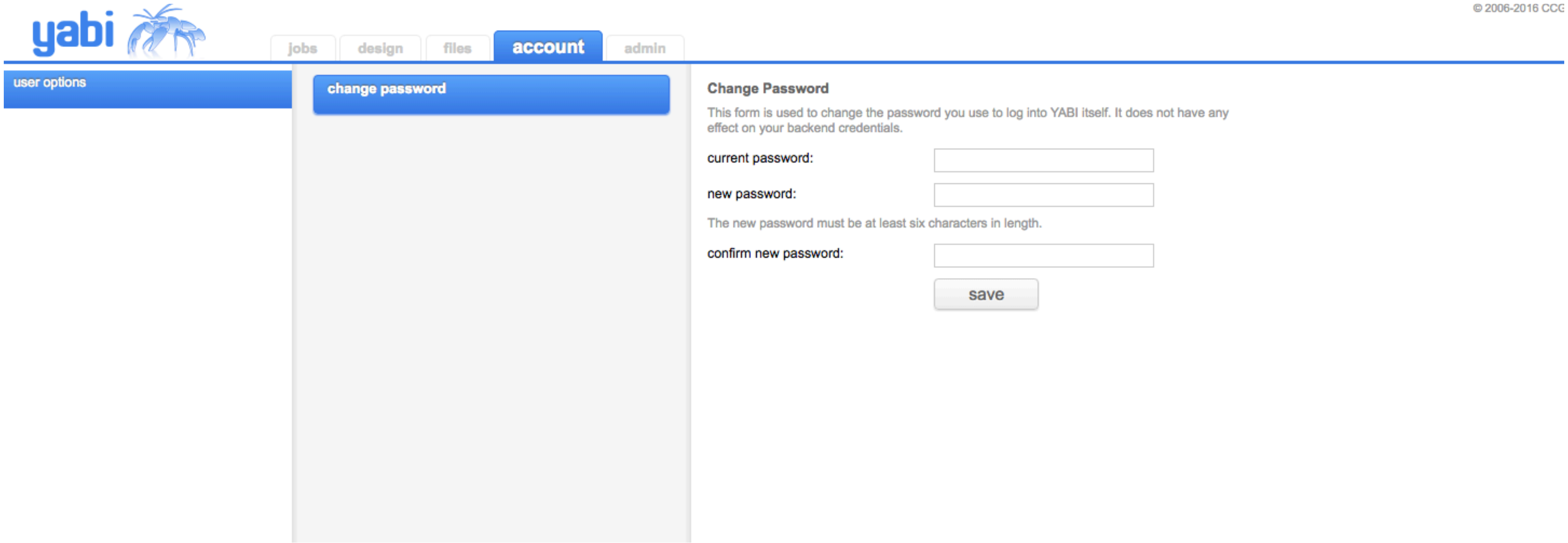

The WorkFlows - Overview

Available pipelines

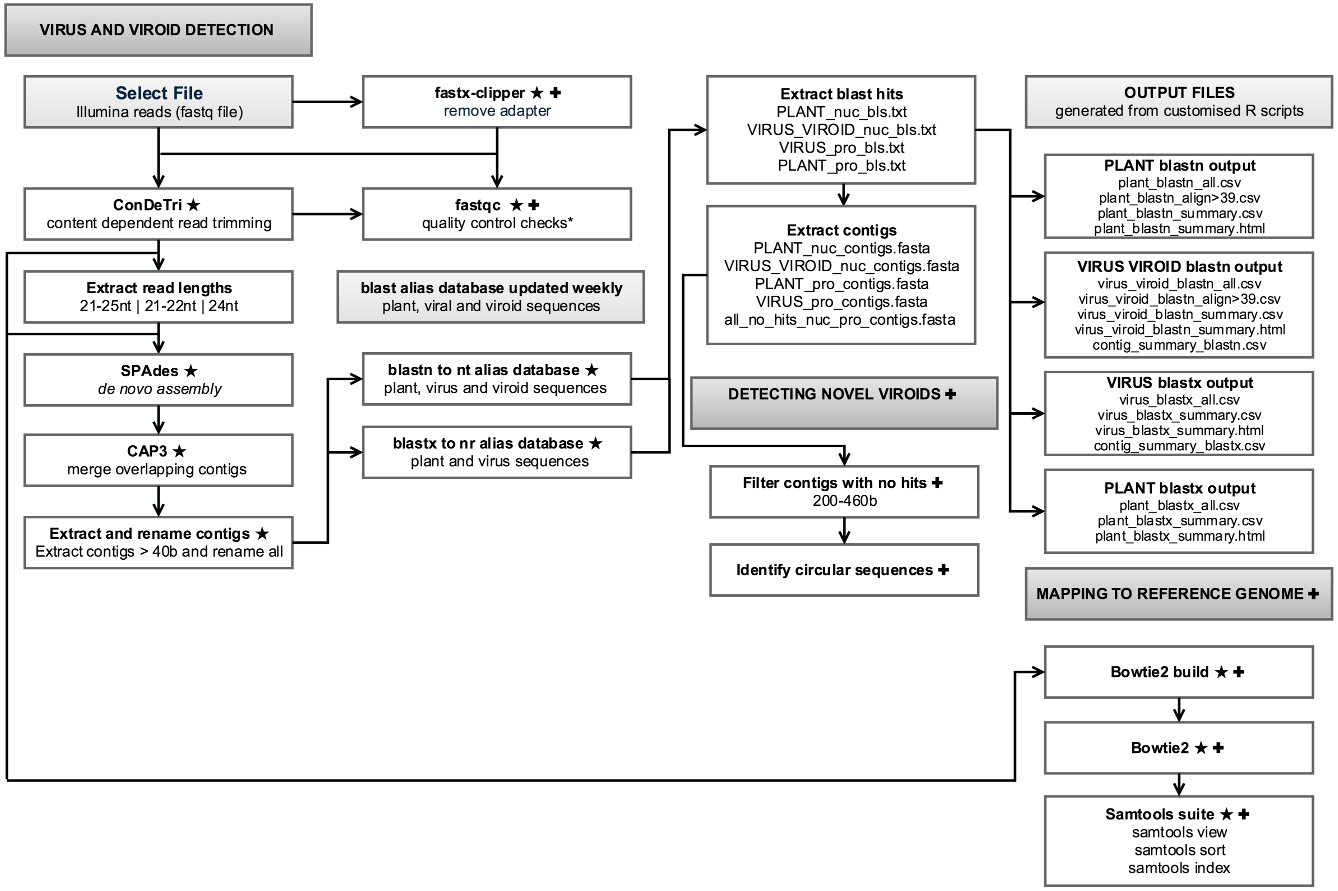

Virus and Viroid Detection and Detection of Novel Viroids

There are three versions of this workflow, utilising:

- 21-25nt reads
- 21-22nt reads
- 24nt reads

The workflows are saved, and accessed from the Design Tab Tools Panel under 'Saved Workflows'. Simply select the workflow of interest, select your file of reads, and run the workflow.

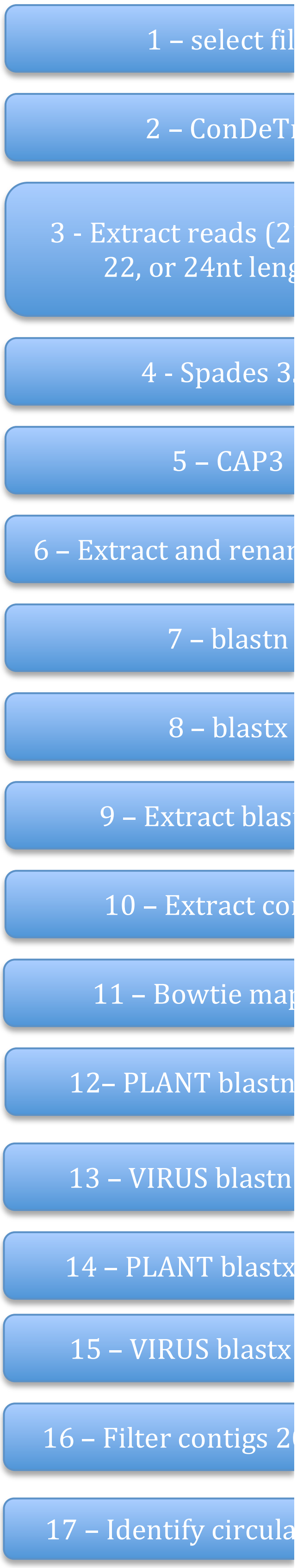

Mapping to Reference Genome

This workflow can be utilised to map reads to viruses or viroids identified in the detection of viruses and viroids workflow.

1 – Select file

2 – Bowtie2 build

3 – Samtools view

4 – Samtools sort

5 – Samtools index

The WorkFlows – Tool Details

The saved workflows are accessed from the Design Tab Tools Panel under 'Saved Workflows'. Simply select the workflow of interest, select your file of reads (Tool 1), and run the workflow.

Virus and Viroid Detection and Detection of Novel Viroids

Tool 1: Select File
Select your FASTQ file from the directory, or upload from your desktop.

Tool 2: ConDeTri
ConDeTri is a content dependent read trimmer for Illumina Data. It trims FASTQ reads from the 3'-end and extract reads (or read pairs) of good quality. If the reads are paired, the filtering is done pairwise, and if one read in a pair has low quality, the remaining read is saved as single end.

Default parameters are used for the following options:

- -hq (high quality threshold, 25)
- -lq (low quality threshold, 10)
- -frac (Fraction of read that must exceed hq, 0.8)
- -mh (When this no of consecutive hq bases is reached, the trimming stops, 5)
- -ml (Max no of lq bases allowed after a stretch of hq bases from 3'-end, 1)
- -cutfirst (Remove i first bases from the 5'end, 0)
- -rmN (Remove non-ATCG bases from 5'end before any trimming, no)

Optimised parameters used for the following options:

- -fastq1 (Fastq(.gz) file, automatically selects from Tool 1, Select File)
- -sc (Illumina scoring table, 33)
- -minlen (Min allowed read length, 18)
- -prefix (Prefix for the output file(s), sample)

Tool 3: Extract reads (21-25nt, 21-22nt, or 24nt)
In house script extracts reads of stated lengths from ConDeTri trimmed reads (automatically selected from Tool 2, ConDeTri).

Tool 4: Spades 3.5 Wrapper
SPAdes is a *de novo* genome assembler that uses k-mers for building the initial de Bruijn graph. It then performs graph-theoretical operations which are based on graph structure, coverage and sequence lengths, and adjusts errors iteratively.

The following options are used:

- -o (directory to store all the resulting files, SPAdes_outpt)

- -memory (memory RAM limit for SPAdes in Gb (terminates if exceeded), 250)
- -k (comma-separated list of k-mer sizes, 15, 17, 19)
- -s (file with unpaired reads, automatically selects from Tool 3, Extract reads).

Tool 5: CAP3 pipeline

CAP3 is a DNA sequence assembly program that is used to assemble overlapping contigs (generated from SPAdes *de novo* assembly). The input file is automatically selected from Tool 4 (Spades 3.5 Wrapper).

Parameters optimised for short reads are used as follows:

- -o (specify overlap length cutoff > 15, 16)
- -p (specify overlap percent identity cutoff N > 65, 90)
- -i (specify segment pair score cutoff N > 20, 30)
- -s (specify overlap similarity score cutoff N > 250, 300)
- -j (specify chain score cutoff N > 30, 31)

Tool 6: Extract and rename contigs

In house script extracts contigs of a minimum length and renames them. This is used as CAP3 (Tool 5) renames overlapping contigs that are assembled.

The following parameters are used:

- -i (input file of contigs, automatically selected from Tool 6 (CAP3 pipeline))
- -n (The minimum number of bases, 40)
- -a (The name of the assembly used to rename the contigs, spades_cap3_CONTIG)

Tool 7: blastn (virus, viroid and plant)

Blastn searches nucleotide databases using a nucleotide query. A custom nucleotide database is created from an in house script that extracts plant, virus and viroid sequences from the blast nt database using the following queries:

- Plant nucleotide: txid33090[orgn]
- Virus nucleotide: txid10239[orgn] NOT txid131567[orgn]
- Viroid nucleotide: txid12884[orgn]

The following blastn parameters are used:

- -query (input file, automatically selected from Tool 6 (Extract and rename contigs))
- -db (BLAST database name, plant, viruses and viroids (as described above))
- -out (output file name, all_nt_bls.txt)
- -evalue (expectation value E, 0.0000000001)
- -task (sets the parameters (e.g., word-size or gap values) to typical values for a specific type of search, blastn-short)

- -outfmt (alignment view options, customised tabular with subject title and query coverage per subject ‘6 qseqid sgi sacc length pident mismatch gapopen qstart qend qlen sstart send slen sstrand evalue bitscore qcovhsp stitle staxids qseq sseq’)
- -max_target_seqs (maximum number of aligned sequences to keep, 5)
- -strand (query strand(s) to search against database/subject, both)

Tool 8: blastx (virus and plant)
Blastx searches protein databases using a translated nucleotide query. A custom protein database is created from an in house script that extracts plant and virus sequences from the blast nr database using the following queries:

- Plant protein: txid33090[orgn]
- Virus protein: txid10239[orgn] NOT txid131567[orgn]

The following blastn parameters are used:
- -query (input file, automatically selected from Tool 6 (Extract and rename contigs))
- -db (BLAST database name, plant and viruses nr (as described above))
- -out (output file name, all_nt_bls.txt)
- -evalue (expectation value E, 0.0000000001)
- -outfmt (alignment view options, customised specified options, tabular ‘6 qseqid sgi sacc length pident mismatch gapopen qstart qend qlen sstart send slen evalue bitscore qcovhsp stitle staxids qseq sseq’)
- -max_target_seqs (maximum number of aligned sequences to keep, 5)
- -strand (query strand(s) to search against database/subject, both)
- -query_gencode (Ggnetic code to use to translate query, standard)

Tool 9: Extract blast hits
In house script extracts blast hits into virus and viroid nucleotide hits, virus protein hits, virus protein hits, and plant nucleotide hits.

An in house script extracts plant, virus and viroid GI numbers from the blast nt and nr databases using the queries described above.

The following options are automatically set:
- -v (viroids and viruses nuc GI)
- -p (plant nuc GI)
- -g (virus pro GI)
- -h (plant pro GI)

The following input files are automatically selected from Tool 7 (blastn) and Tool 8 (blastx)
- -n (all_nt_bls.txt)
- -r (all_nr_bls.txt)

Tool 10: Extract contigs- plant, virus, no hits
An in house script extracts the contigs from blast hits.

The following input files are automatically selected from various tools:

- -f (all_contigs_rename.fasta, automatically selected from Tool 6 (extract and rename contigs))
- -p (PLANT_nuc_bls.txt, automatically selected from Tool 9 (Extract blast hits))
- -v (VIRUS_VIROID_nuc_bls.txt, automatically selected from Tool 9 (Extract blast hits))
- -g (PLANT_pro_bls.txt, automatically selected from Tool 9 (Extract blast hits))
- -a (all_nt_bls.txt, automatically selected from Tool 7 (blastn))
- -b (all_nr_bls.txt, automatically selected from Tool 8 (blastx))

Tool 11: Bowtie mapping
An in house script maps the FASTQ file of input reads (taken from the raw file of reads (Tool 1), or ConDeTri qc'd reads (Tool 2), or read lengths of various sizes (Tool 3)) to the contigs that have hits to virus sequences from blastn and blastx (VIRUS_VIROID_nuc_contigs.fasta and VIRUS_pro_contigs.fasta, Tool 10).

Tool 12: PLANT blastn output
An in house script summarises the plant blastn output. The input file is (PLANT_nuc_bls.txt) is automatically selected from Tool 9 (Extract blast hits).

Tool 13: VIRUS blastn output
An in house script summarises the virus and viroid blastn output. The input file (VIRUS_VIROID_nuc_bls.txt) is automatically selected from Tool 9 (Extract blast hits).

Tool 14: PLANT blastx output
An in house script summarises the plant blastx output. The input file is (PLANT_pro_bls.txt) is automatically selected from Tool 9 (Extract blast hits).

Tool 15: VIRUS blastx output
An in house script summarises the virus blastx output. The input file (VIRUS_pro_bls.txt) is automatically selected from Tool 9 (Extract blast hits).

Tool 16: Filter contigs 200-460nt
An in house script filters contigs with no hits to nucleotide or protein sequences, that are between 200-460 nt in length. The input file (all_no_hits_nuc_pro_contigs.fasta) is automatically selected from Tool 10 (Extract contigs- plant, virus, no hits).

Tool 17: Identify circular contigs
An in house script identifies circular contigs that may indicate novel viroid sequences. Input file (contigs_no_hits_200-460.fasta) is automatically selected from Tool 15 (Filter contigs 200-460nt).

Additional screening of any contigs identified with tools such as RFAM, miRBase, tRNAscaen-SE and RNAfold is recommended.

Mapping to Reference Genome

Tool 1: Select File
Upload the FASTA file of the genome of interest (obtained from NCBI). Select your FASTQ/FASTA file of reads from the directory, or upload from your desktop.

Tool 2: bowtie-build
Bowtie[1] is an ultrafast, memory-efficient short read aligner geared toward quickly aligning large sets of short DNA sequences (reads) to large genomes. Bowtie-build builds the index of the FASTA file of the genome of interest.

The following parameters are used:

- inputFile (FASTA file of the genome of interest is automatically selected from Tool 1: Select File)
- outfileBase (The base string to use for the output files, outfile_base)

Tool 3: bowtie
Bowtie aligns the reads to the genome of interest.

The following parameters are used:

- -q (query input files are FASTQ .fq/.fastq, yes)
- -v (3)
- -m (suppress all alignments if > n exist, 1)
- --best (hits guaranteed best stratum; ties broken by quality, yes)
- -S (write hits in SAM format, yes)
- ebwt (The basename of the index for the reference genome, outfile_base)
- -indexes (Dummy parameter to select bowtie2 index files, automatically selected from Tool 2: bowtie-build)

Tool 4: samtools view
SAMtools[2] provide various utilities for manipulating alignments in the SAM format, including sorting, merging, indexing and generating alignments in a per-position format.

The following parameters are used:

- -o Output file (e.g. *.bam, outfile_base)

- InputFile (Input File in SAM or BAM format, automatically selected from Tool 3 (bowtie)
- -b (Output bam file, yes)

Tool 5: samtools sort

The following parameters are used:

- InputFile (Input file to sort, automatically selected from Tool 4 (samtools view)
- outPutPrefix (Prefix to use in file name, sorted)

Tools 6: samtools index

The following parameters are used:

- InputFile (Input file in bam format, automatically selected from Tool 5 (samtools index)

The WorkFlows – Output Files Details

To obtain the result files for each workflow, select the workflow of interest from the Jobs tab. Click on the Tool of interest in the middle (workflow) panel and then preview or download the output file of interest from the right (output) panel. To preview the output file, click on the file name or blue magnifying glass in the 'file outputs tab'. While previewing the output file, you may also download or navigate to the preview of the next or previous output files. To download, click on the grey down arrow. To navigate to the preview of the previous or next output file, click on the left or right arrows. To exit out of the preview screen, click the grey cross.

To download the output file from the file outputs tab, click on the green arrow. Depending on the web browser or operating system in use, the file may download directly to your web browser and downloads folder, or you may receive an option to save or open the file in a relevant program (such as excel for a .csv file).

Files that are 0.0 kB in size are empty. Files ending in '.o' or '.po' contain verbose details as Tools progress (not all Tools record verbose output). Files ending in '.e' or '.pe' contain error messages resulting from the Tool.

Virus and Viroid Detection and Detection of Novel Viroids

Tool 1: Select File

- File of reads selected

Tool 2: ConDeTri

- Sample.stats contains basic statistics:
    - Number of reads in original file
    - Number of bases original file
    - Number of paired reads after trimming
    - Number of bases in pairs after trimming
    - Number of unpaired reads after trimming
    - Number of unpaired bases after trimming
- Sample_trim.fastq contains the reads retained after trimming

Tool 3: Extract reads (21-25nt, 21-22nt, or 24nt)

- *nt_reads.fq contains the reads retained of the relevant length (21-25nt, 21-22nt, or 24nt)

Tool 4: Spades 3.5 Wrapper

- contigs.fasta contains the contigs produced from SPAdes. This is the file used in additional steps of the workflow
- SPAdes_output directory contains all output pertaining to the SPAdes tool
- Output files in the main directory are the combined kmer results

- Individual kmer directories (such as K15, K17, K19) contain the output files for individual kmer runs
- corrected/ directory contains reads corrected by BayesHammer in *.fastq.gz files; if compression is disabled, reads are stored in uncompressed *.fastq files
  - configs – configuration files for read error correction
  - corrected.yaml – internal configuration file
- before_rr.fasta and before_rr.fastg - contigs before repeat resolution
- contigs.fasta contains resulting contigs
- contigs.fastg resulting contigs in FASTG format
- dataset.info – internal configuration file
- input_dataset.yaml – internal YAML data set file
- params.txt – information about SPAdes parameters in this run
- scaffolds.fasta contains resulting scaffolds
- scaffolds.fastg resulting scaffolds in FASTG format
- spades.log – SPAdes log
- warnings.log – warnings log

Tool 5: CAP3 pipeline

- all_contigs.fasta contains the resulting contigs from CAP3 (combined contigs and singles)

Tool 6: Extract and rename contigs

- all_contigs_rename.fasta contains the renamed contigs

Tool 7: blastn (virus, viroid and plant)

The following blastn parameters are used:

- all_nt_bls.txt contains the blast output in tabular format

Tool 8: blastx (virus and plant)

- all_nr_bls.txt contains the blast output in tabular format

Tool 9: Extract blast hits

- PLANT_nuc_bls.txt – blastn results with hits to plant sequences
- PLANT_pro_bls.txt – blastx results with hits to plant sequences
- VIRUS_VIROID_nuc_bls.txt – blastn results to virus and viroid sequences
- VIRUS_pro_bls.txt – blastx results to virus sequences

Tool 10: Extract contigs- plant, virus, no hits

- PLANT_nuc_contigs.fasta – contigs with blastn hits to plant sequences
- PLANT_pro_contigs.fasta – contigs with blastx hits to plant sequences
- VIRUS_VIROID_nuc_contigs.fasta – contigs with blastn hits to virus and viroid sequences

- VIRUS_pro_contigs.fasta – contigs with blastx hits to virus sequences
- all_hits_nuc_pro_contigs.fasta – contigs with blastn and blastx hits to plant, virus and viroid sequences
- all_no_hits_nuc_pro_contigs.fasta – contigs with no hits to plant and virus sequences from blastn and blastx

Tool 11: Bowtie mapping output

- nuc_contigs_mapped_reads_number.txt
- pro_contigs_mapped_reads_number.txt

Output tools:

- The following tools produce the output files of most interest:
- *summary.csv and *summary.html files summarise the virus, viroid or plant sequences detected, the number of contigs hits, alignment lengths, average % ID, and % coverage by contigs. The *.html files provide ease of viewing results within Yabi. The *.csv files can be downloaded and opened in Excel.
    - The total contig lengths (summed) and estimated potential contig coverage (%) columns are estimates only, designed to provide additional information on the length of contig versus the alignment length. For example, if a contig has an alignment to a viral sequence that is 80b, but the contig itself is 2000b in length, it may indicate that the virus is not truly present. Additionally, it may highlight a novel virus.
    - Note that the total contigs summed may include the same contig length added together in cases of multiple hits to a particular viral sequence by the same contig
- Note that the detection of a particular virus/viroid sequence detailed in this file does not necessarily indicate presence of the virus/viroid. For example, one contig may have hits to multiple, closely related, virus or viroid sequences (the top 5 hits are reported in blast output).
- contigs_summary_blast*.csv files summarise the details of contigs with blast hits. Use this file to check if a contig has hits to multiple viruses/viroids in order to determine if a virus/viroid is present.
- *blast*_all.csv files are the same blast results in tabular format (from Tools 7 or 8), but with a header added for explanation
- *blastn_align>39b.csv is a subset of results (only reports alignments of greater than 39b in length) from the *blastn_all.csv files. This file is then used to produce the *_blastn_summary.csv and *_blastn_summary.html files.

Tool 12: PLANT blastn output

- plant_blastn_all.csv
- plant_blastn_align>39b.csv
- plant_blastn_summary.csv

- plant_blastn_summary.html

Tool 13: VIRUS blastn output
Note: this output reports both viruses and viroids

- virus_blastn_all.csv
- virus_blastn_align>39b.csv
- virus_blastn_summary.csv
- virus_blastn_summary.html
- contig_summary_blastn.csv

Tool 14: PLANT blastx output

- plant_blastx_all.csv
- plant_blastn_summary.csv
- plant_blastn_summary.html

Tool 15: VIRUS blastx output

- virus_blastx_all.csv
- virus_blastx_summary.csv
- virus_blastx_summary.html
- contig_summary_blastx.csv

Tool 16: Filter contigs 200-460nt

- contigs_no_hits_200-460.fasta – this file contains contigs that have no hits from blastn or blastx that are 200-460b in length

Tool 17: Identify circular contigs

- circular_contigs.fasta – contains potential novel viroid sequences. SPAdes produces 'circular' contigs that have hits to viroid sequences. Additional screening of any contigs identified with tools such as RFAM, miRBase, tRNAscaen-SE and RNAfold is recommended.

Mapping to Reference Genome

Tool 1: Select File

- FASTA/FASTQ files selected

Tool 2: bowtie-build

- *.ebwt – the bowtie build reference index files

Tool 3: bowtie

- *.sam – the file containing the details of the mapping of reads to the contigs

Tool 4: samtools view

- *.bam – conversion of the *.sam file into *.bam format for sorting

Tool 5: samtools sort

- *sorted.bam – alignments are sorted by leftmost coordinates

Tools 6: samtools index

- *sorted.bam.bai – Indexes the coordinate-sorted BAM file for fast random access.

References

1. Langmead, B. and S.L. Salzberg, *Fast gapped-read alignment with Bowtie 2.* Nature methods, 2012. **9**(4): p. 357-359.
2. Li, H., et al., *The sequence alignment/map format and SAMtools.* Bioinformatics, 2009. **25**(16): p. 2078-2079.
