# Supplementary figures and images for: An internet-based bioinformatics toolkit for plant biosecurity diagnosis and surveillance of viruses and viroids
Source: BMC Bioinformatics. 2017 Jan 11;18:26. doi: 10.1186/s12859-016-1428-4 (PMC5225587; doi:10.1186/s12859-016-1428-4)

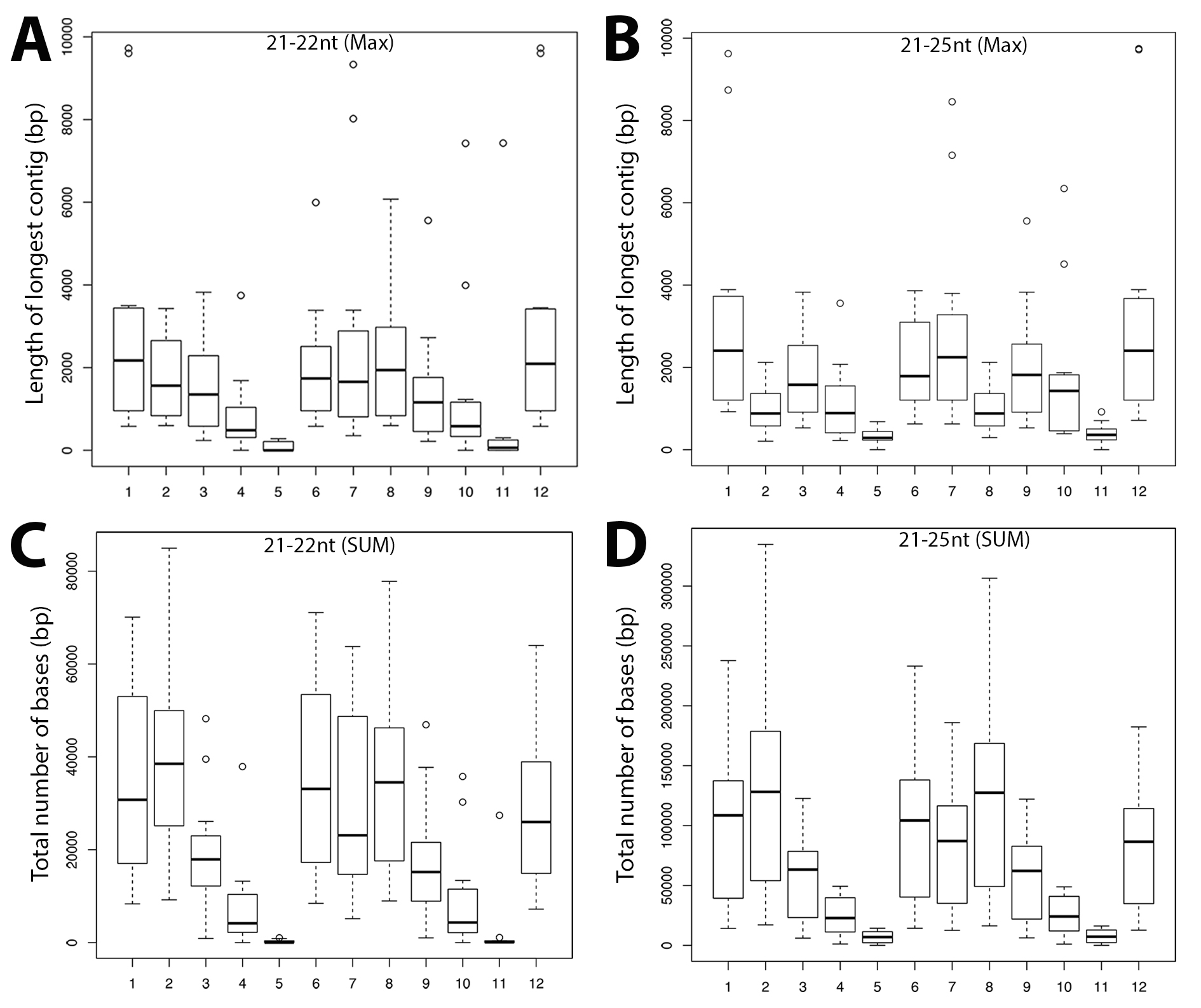

Supplement: Additional file 3: — Boxplot comparison of SPAdes assemblies using individual kmers, sets of kmers and CAP3 scaffolding. (JPG 527 kb) [file 12859_2016_1428_MOESM3_ESM.jpg]

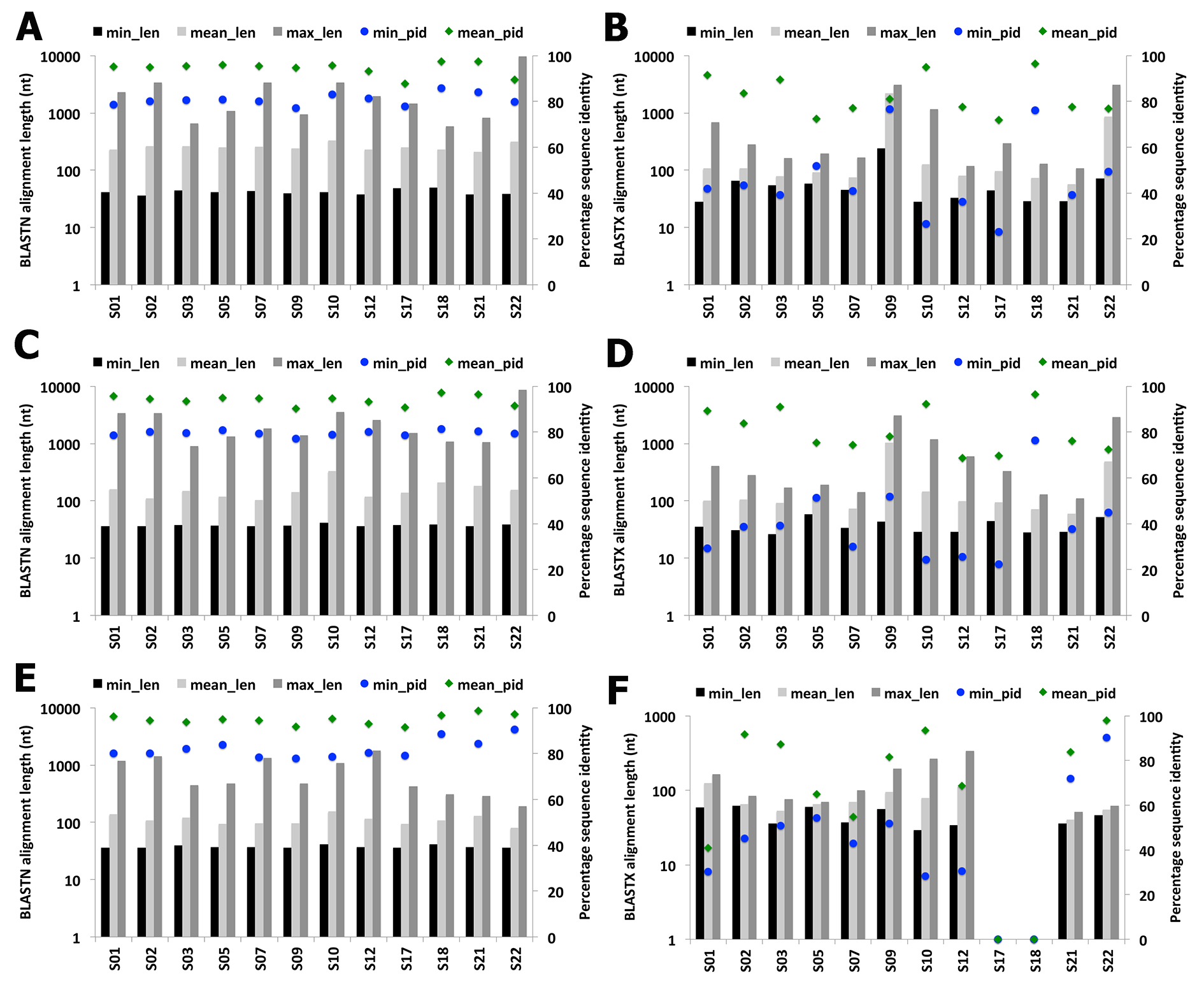

Supplement: Additional file 5: — BLASTN and BLASTX sequence similarity statistics for de novo assembled contigs using the 21–22 nt (A and B), 21–25 nt (C and D) and 24 nt (E and F) pipelines, respectively. Twelve small RNA-Seq samples generated in this study with nucleotide similarity to viral pathogens were compared. See Additional file 1 for details of the selected samples. (JPG 809 kb) [file 12859_2016_1428_MOESM5_ESM.jpg]

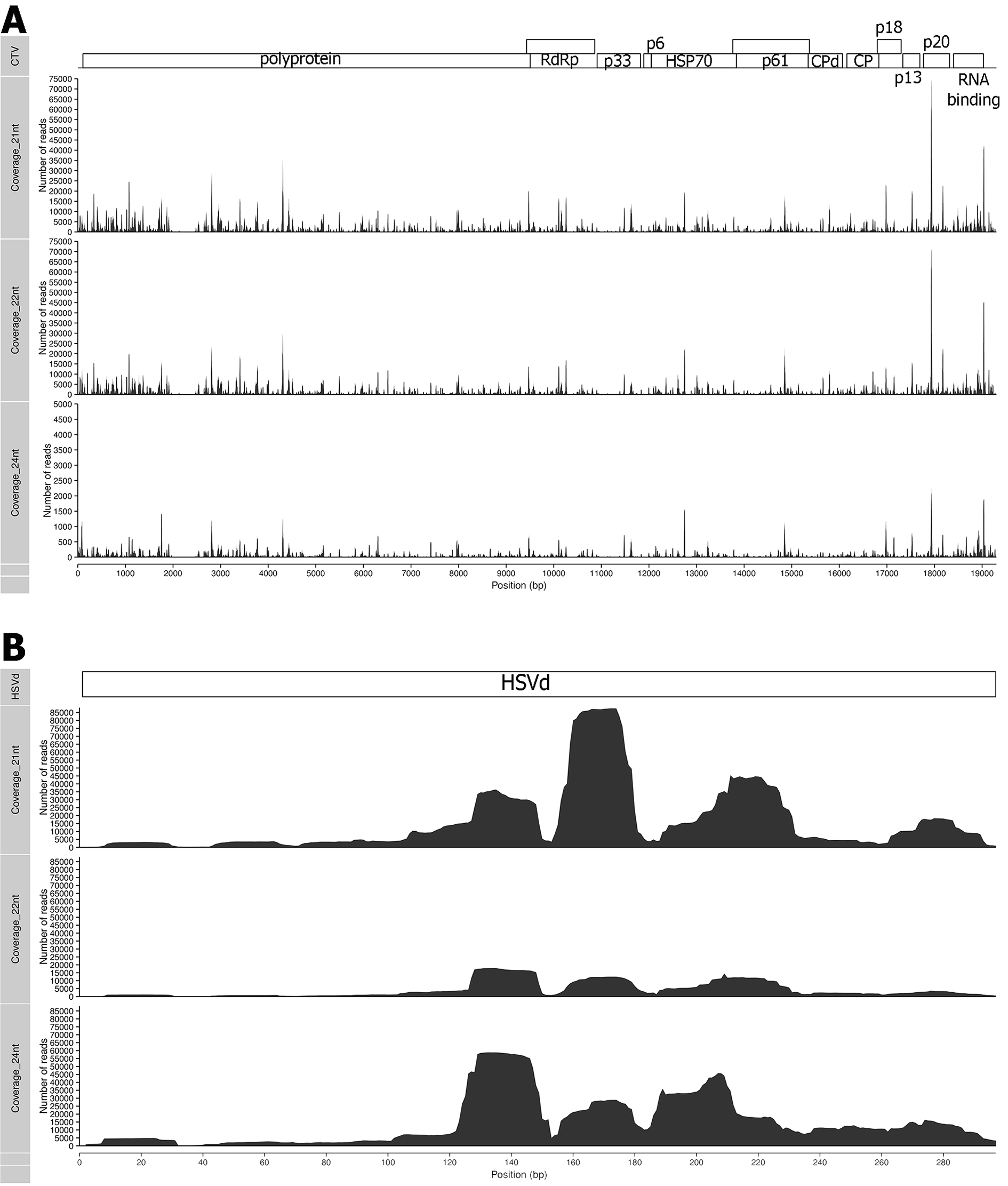

Supplement: Additional file 7: — Mapping of high quality adaptor-clipped and quality trimmed small RNAs with no mismatches onto the reference genomes: A) Citrus tristesa virus (CTV; accession number AB046398). Domains of the CTV genomes are denoted. B) Hop Stunt Viroid (HSVd). (JPG 702 kb) [file 12859_2016_1428_MOESM7_ESM.jpg]

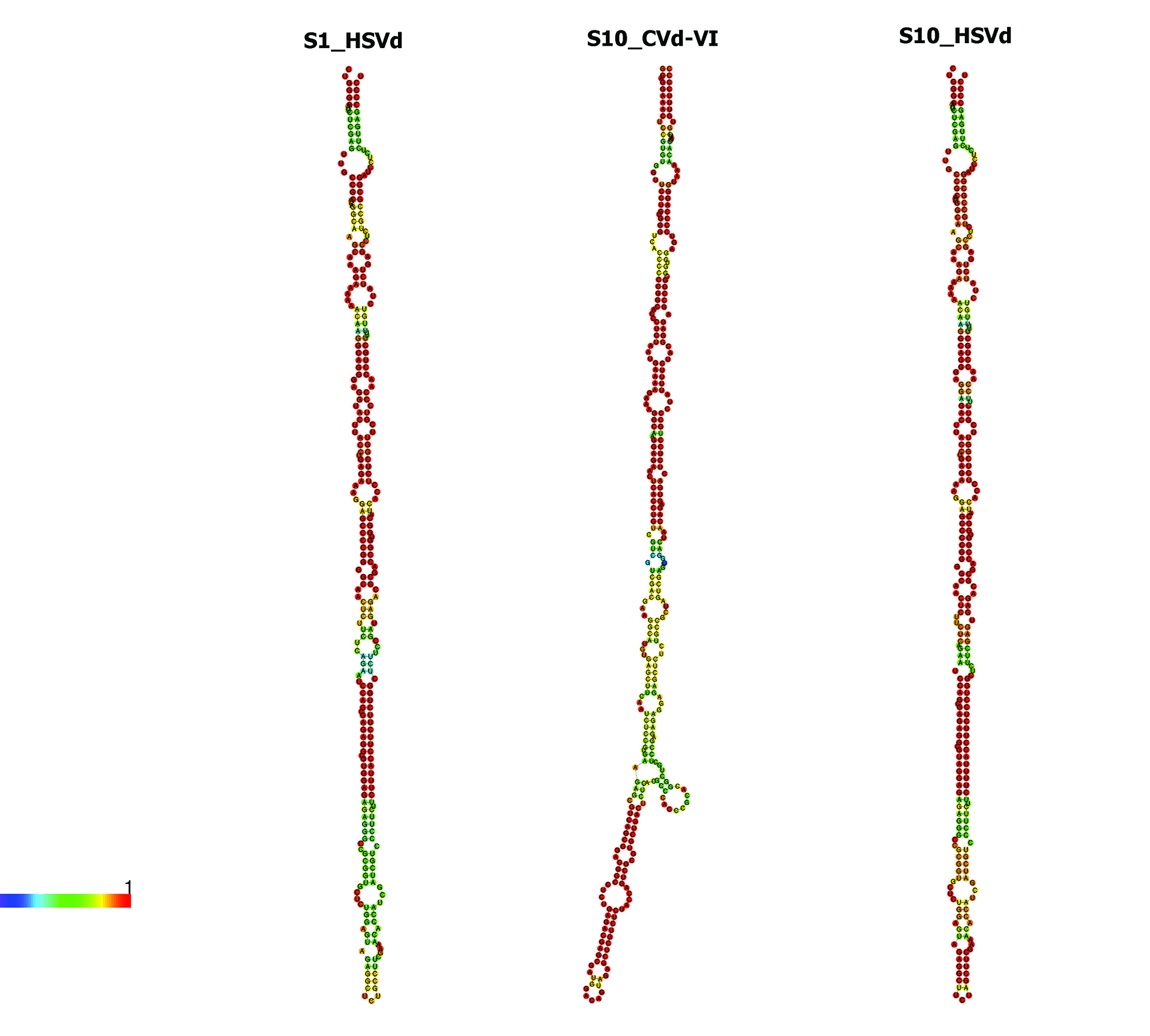

Supplement: Additional file 8: — Predicted RNA secondary structure of viroids found in this study. Minimal free energy RNA secondary structure encoding base-pair probabilities are shown for S1_HSVd (sample 1 – Hop Stunt Viroid), S10_CVD-VI (sample 10 – Citrus Viroid VI), and S10_HSVd (sample 10 – Hop Stunt Viroid). (JPG 1406 kb) [file 12859_2016_1428_MOESM8_ESM.jpg]

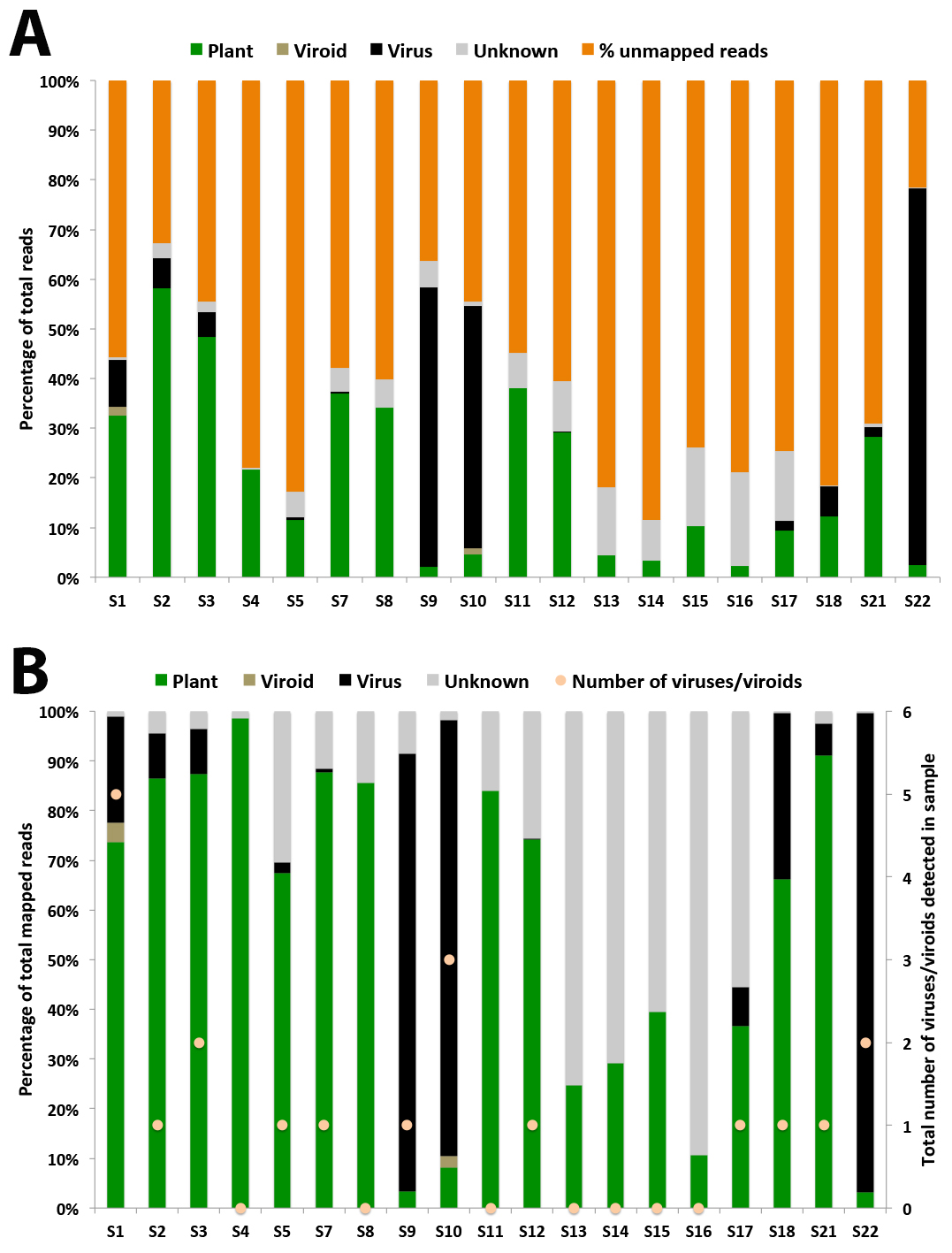

Supplement: Additional file 9: — Distribution of mapped and unmapped small RNA reads for quarantined samples generated in this study. A) Percentage of unmapped and mapped reads onto de novo assembled contigs with sequence similarity to plants; viruses, viroids and unknown are shown. B) Distribution of the percentage of mapped reads for each sample along with the total number of distinct viral sequences (viruses/viroids) are shown. Details for each sample can be found in Additional file 1. (JPG 477 kb) [file 12859_2016_1428_MOESM9_ESM.jpg]

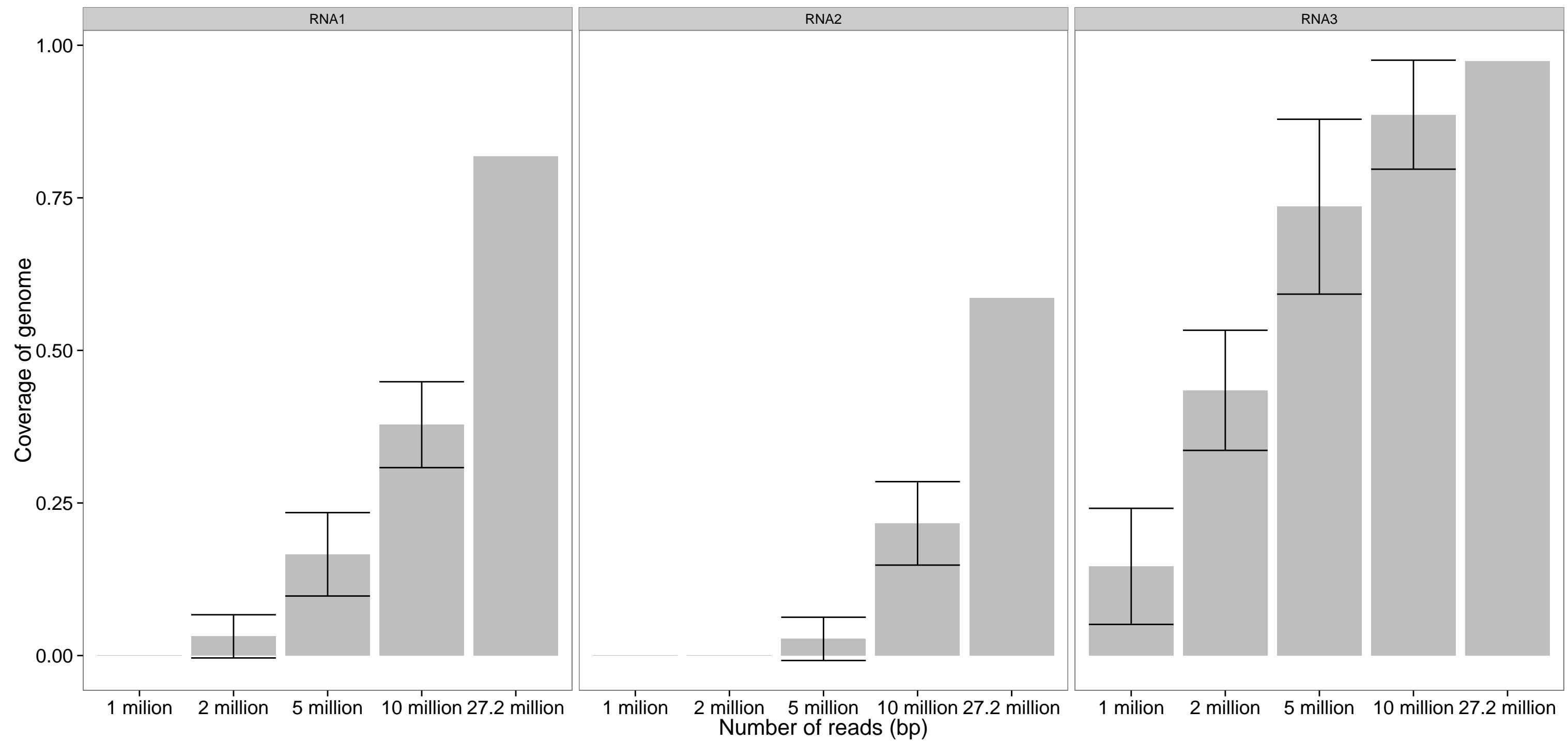

Supplement: Additional file 10: — De novo assembly of PNRSV RNA1, RNA2 and RNA3 viral sequences using five randomly generated subsets of 1 M, 2 M, 5 M, 10 M and all adaptor clipped small RNA reads. (PDF 5 kb) [file 12859_2016_1428_MOESM10_ESM.pdf]

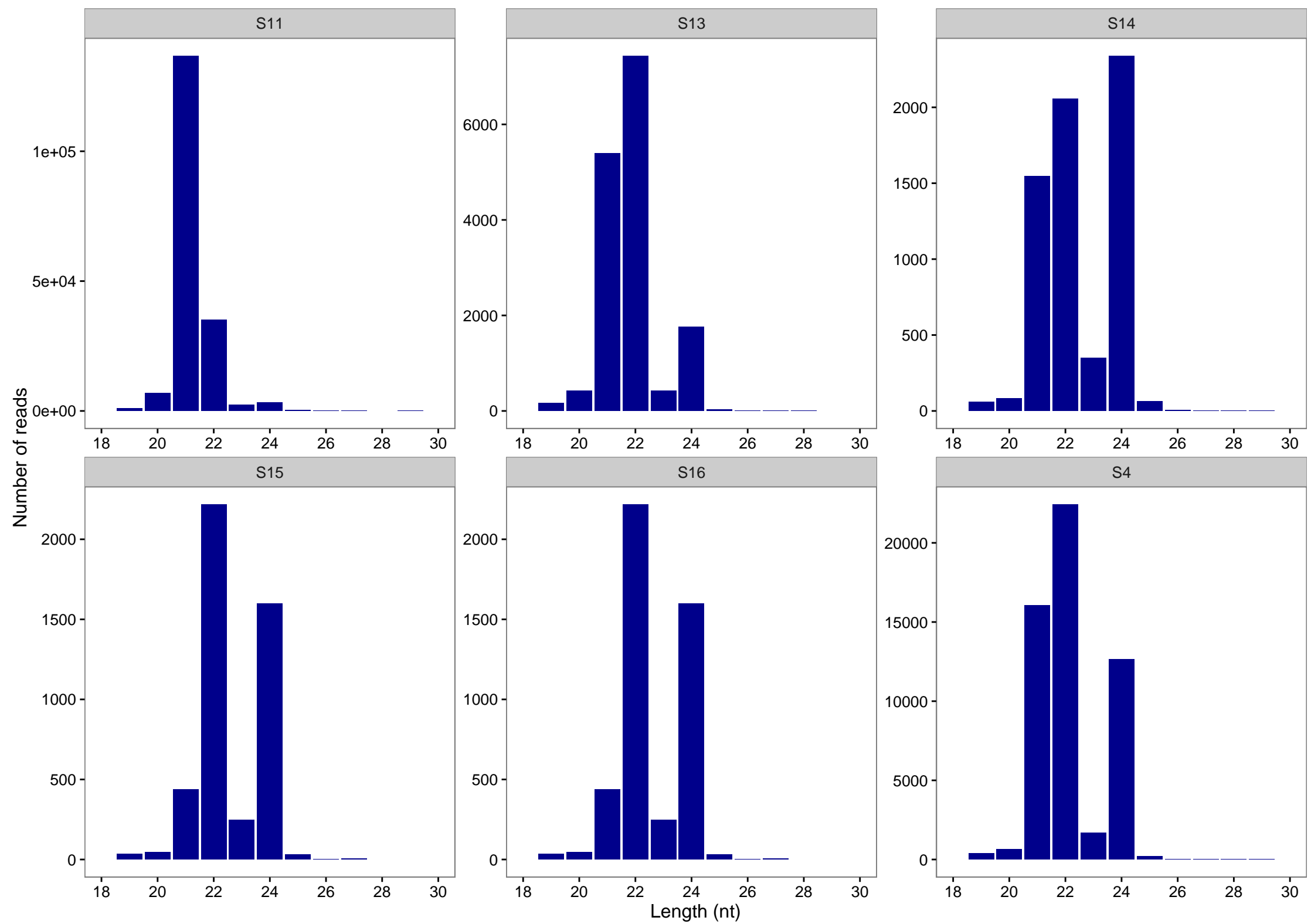

Supplement: Additional file 13: — Mapping of adaptor clipped reads onto VirFind assembled contigs for samples S4, S11, S13, S14, S15 and S16. Details of samples can be found in Additional file 1. (PDF 5 kb) [file 12859_2016_1428_MOESM13_ESM.pdf]

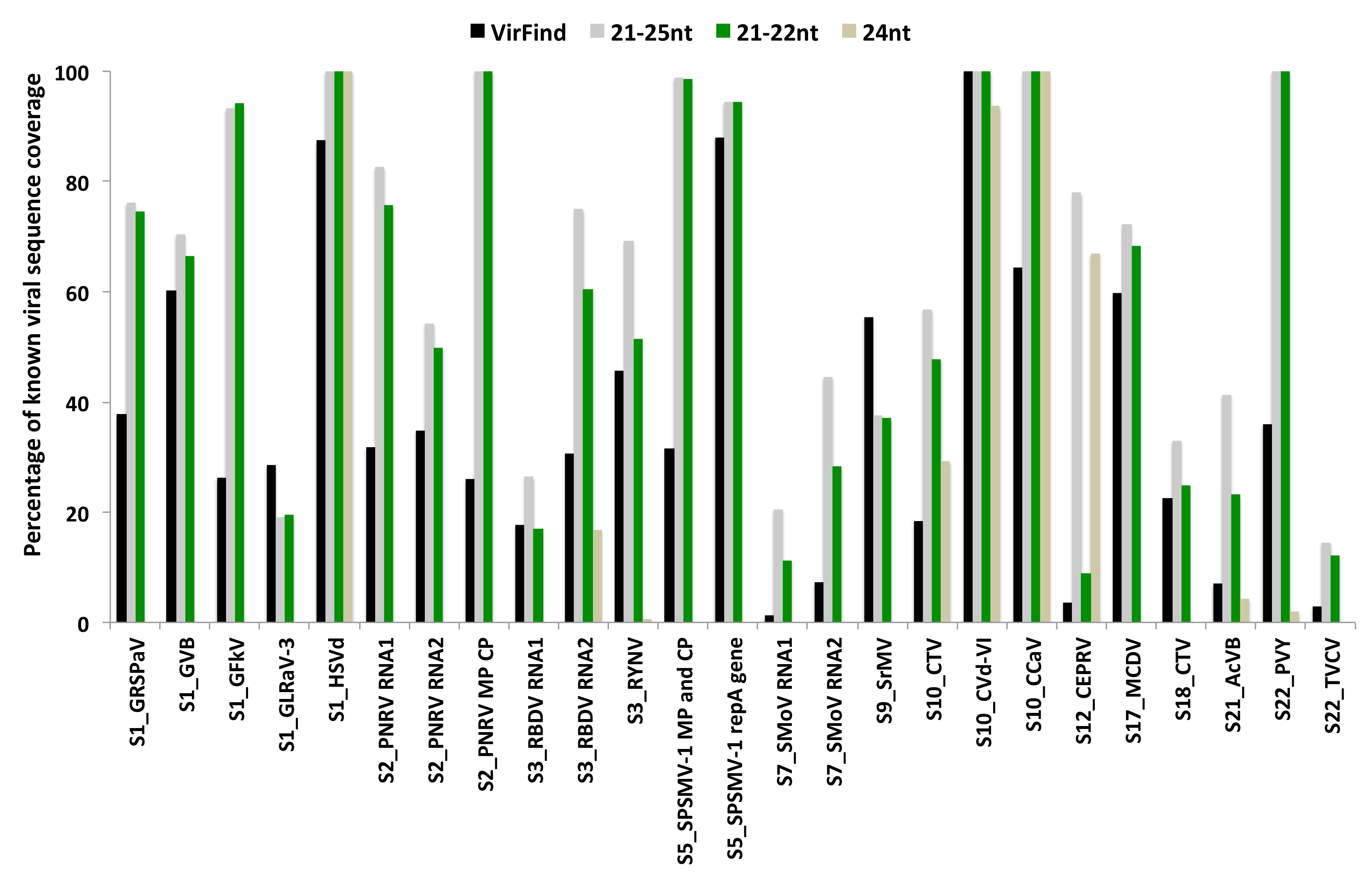

Supplement: Additional file 15: — Comparison of genome coverage of known viral sequences by contigs assembled by the VSD toolkit and VirFind analysis pipelines. (JPG 1197 kb) [file 12859_2016_1428_MOESM15_ESM.jpg]
